# Radiation-induced peroxide rupture and its temperature-dependent repair probed by homogeneous X-ray irradiation

**DOI:** 10.1107/S2059798326002688

**Published:** 2026-04-13

**Authors:** Symeon Koulas, Soi Bui, Julius B. Kirkegaard, Gleb Bourenkov, Roberto A. Steiner

**Affiliations:** a Department of Biomedical Sciences University of Padova Padova Italy; b Randall Centre for Cell and Molecular Biophysics King’s College London London United Kingdom; c Niels Bohr Institute and Department of Computer Science, Copenhagen, Denmark; d European Molecular Biology Laboratory Hamburg Unit c/o DESY Hamburg Germany; Institut de Biologie Structurale, France

**Keywords:** radiation damage, enzymology, reaction kinetics, atomic resolution

## Abstract

Specific radiation damage frequently compromises structural analysis in macromolecular crystallography. However, it can also offer key mechanistic information. Here, we investigate the X-ray-induced radiolysis of the catalytic C5-peroxide adduct in crystals of the cofactor-independent enzyme urate oxidase. Using a top-hat X-ray beam to ensure homogeneous dose distribution, we monitored the occupancy of the peroxide species across extensive dose series at 100 K and room temperature (RT). We observe a fundamental kinetic phase transition between these thermal regimes. At RT, the peroxide decays rapidly following zero-order kinetics, consistent with a flux-limited regime where the radiolytically cleaved O_2_ product diffuses out of the active site to be replaced by water. Conversely, at 100 K the decay is markedly retarded and follows first-order kinetics. Bayesian kinetic modelling demonstrates that this cryoprotection arises from a recombination mechanism: the cleaved O_2_ molecule remains trapped in the active site with the organic species, enabling efficient recombination that competes with irreversible degradation, effectively resulting in radiation-induced *in crystallo* catalysis.

## Abstract 2

This manuscript investigates the X-ray-induced radiolysis of the catalytic C5-peroxide adduct in urate oxidase crystals, utilizing a top-hat X-ray beam to monitor peroxide occupancy across extensive dose series at both 100 K and room temperature. The study reveals a kinetic phase transition where the peroxide decays rapidly via zero-order kinetics at room temperature, whereas at 100 K the decay is retarded and follows first-order kinetics due to an efficient recombination mechanism involving trapped oxygen.

## 1. Introduction

Since the earliest days of macromolecular X-ray crystallo­graphy (MX), radiation-induced damage to crystalline samples has been recognized as a critical experimental factor (Blake & Phillips, 1962 ▸). When X-ray photons impinge on a crystal, only a small portion interacts with matter (∼2% in the 5–17 keV range typically used in MX). Of these interactions, ∼8% contribute to elastic scattering and the desired diffraction pattern, ∼8% involve inelastic scattering with energy transfer and the remaining ∼84% lead to photoelectric excitation (Ravelli & Garman, 2006 ▸). Primary photoelectrons have a pathlength of a few micrometres and in small crystals some escape before depositing their full energy, thereby reducing potential damage (Cowan & Nave, 2008 ▸; Nave & Hill, 2005 ▸). While still in the crystal, however, these electrons can undergo inelastic scattering with surrounding atoms, generating cascades of lower energy secondary electrons and creating positively charged centres (secondary damage).

The total dose, defined as the amount of energy absorbed per unit mass of crystal (1 Gy = 1 J kg^−1^), is determined both by the photon absorption characteristics of the crystal, which depend on its chemical composition, and by the number of incident photons. A major advance in minimizing radiation damage in MX was the introduction of cryocooling (Hope, 1988 ▸). At 100 K, the lifetime of a crystal is extended by approximately a factor of 70 compared with room-temperature (RT) experiments due to the reduced mobility of radiation-induced free radicals (Nave & Garman, 2005 ▸).

At a global level, radiation damage manifests itself in reciprocal space as a decrease in the average diffraction intensity, a worsening of merging statistics and an increase in the Wilson *B* factor (Garman & Weik, 2017 ▸). Specific damage to the most radiation-sensitive regions in macromolecules can also occur. Well known examples include the rupture of disulfide bonds, cleavage of the CG–S bond in methionine residues and the decarboxylation of Asp/Glu side chains (Fioravanti *et al.*, 2007 ▸; Ravelli & McSweeney, 2000 ▸; Weik *et al.*, 2000 ▸; Burmeister, 2000 ▸), as well as the reduction of redox-active metal centres (Bolton *et al.*, 2024 ▸; Yano *et al.*, 2005 ▸). Radiation-induced breakage of covalent bonds in nucleic acids has likewise been observed, such as cleavage of the C—Br bond in brominated DNA crystals (McGeehan *et al.*, 2007 ▸).

The analysis of radiation-damage effects in MX, particularly specific ones, must also carefully consider possible beam-shape effects. Nonhomogeneous irradiation within the crystal volume by a Gaussian X-ray beam can lead to a ‘hole-burning’ effect whereby different regions of the sample decay at different rates (Warkentin *et al.*, 2017 ▸). This can complicate the interpretation of electron-density maps, as shown for the apparent dose-dependent reversal of radiation-damaged disulfide bridges in crystals of hen egg-white lysozyme (Carpentier *et al.*, 2010 ▸; de la Mora *et al.*, 2020 ▸).

Urate oxidase (UOX) is a ∼150 kDa enzyme responsible for the O_2_-dependent degradation of uric acid to 5-hydroxy­isourate (5-HIU) (Imhoff *et al.*, 2003 ▸; Kahn & Tipton, 1998 ▸; Wei *et al.*, 2017 ▸; Mori *et al.*, 2023 ▸). Although UOX is present across all three domains of life, humans lack this enzyme as its pseudogenization during primate evolution led to inactivation of the uricolytic pathway (Oda *et al.*, 2002 ▸). For this reason, recombinant UOX (rasburicase) is used therapeutically for the treatment of severe hyperuricemia and to prevent the surge in UA levels associated with tumour lysis after certain chemotherapeutic treatments (Cheuk *et al.*, 2017 ▸).

**Figure 1:**
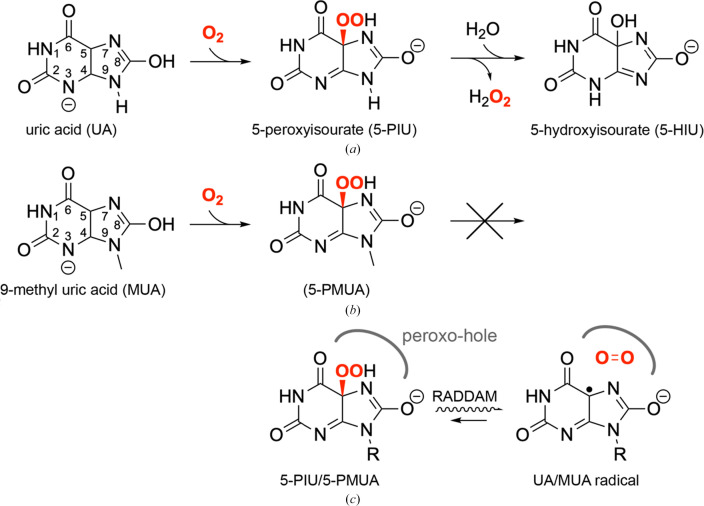
UOX-catalysed reaction and peroxide radiolysis. (*a*) UOX catalyses the O_2_- and H_2_O-dependent conversion of uric acid (UA), present predominantly in its monoanionic form at physiological pH, to 5-hydroxyisourate (5-HIU). The first step of the reaction proceeds via formation of the 5-peroxyisourate (5-PIU) intermediate. (*b*) Chemical structure of 9-methyl uric acid (MUA), the alternative substrate used in this work. MUA undergoes oxygenation to form its 5-peroxy derivative [5-hydroperoxy-(9-methyl)-8-oxy-5,9-dihydro-1*H*-purine-2,6-dione, 5-PMUA], but unlike 5-PIU it is unable to advance further in the catalytic mechanism as it lacks the hydrogen at position 9. Here and in (*a*), the five-membered ring of the substrate is shown in its lactim form as indicated by neutron diffraction studies of the aerobic chloride-inhibited UOX–UA complex (Oksanen *et al.*, 2014 ▸). (*c*) Both peroxides (the *R* substituent at position 9 is H in UA, whilst it is CH_3_ in MUA) are easily radiolysed during X-ray data collection. Following radiation-induced damage (RADDAM) under cryogenic conditions, O_2_ remains trapped in the ‘peroxo-hole’ in close proximity to the organic moiety, which is believed to be a stable UA/MUA radical. A mechanism of partial 5-PMUA regeneration is believed to be operational alongside the RADDAM process (Bui *et al.*, 2014 ▸). In all panels, the C5—OO(H) bond that is selectively broken/formed is shown in red.

In previous work, we unambiguously showed that the first step in UOX-mediated degradation of UA involves the formation of a C5-peroxide intermediate (5-PIU), which then converts to 5-HIU through sequential H_2_O_2_ elimination and hydration (Fig. 1 ▸*a*; Bui & Steiner, 2016 ▸; Bui *et al.*, 2014 ▸; McGregor *et al.*, 2021 ▸). We also showed that the 9-methyl derivative of UA (MUA) reacts with O_2_ to form a similar C5-peroxide (5-PMUA), which is longer lived as it cannot proceed further along the catalytic pathway (Fig. 1 ▸*b*). Both peroxides are remarkably sensitive to radiolysis, and using MX supported by *in crystallo* Raman spectroscopy at cryogenic temperature we demonstrated that very low X-ray doses specifically cleave the C5—OO(H) bond, releasing O_2_*in situ*, which becomes trapped in the ‘peroxo-hole’ above the radiolysed organic moiety (Bui *et al.*, 2014 ▸; Fig. 1 ▸*c*). UV–Vis absorption measurements indicated that the resulting species is likely a resonance-stabilized urate radical (Bui *et al.*, 2014 ▸) that has been shown to be unreactive toward ground-state O_2_ (Simic & Jovanovic, 1989 ▸).

From the dose-dependent rate of bond rupture, we previously proposed that at cryogenic temperatures a mechanism of peroxide regeneration operates alongside peroxide decomposition (Bui & Steiner, 2016 ▸; Bui *et al.*, 2014 ▸). To further investigate this, we collected dose-series datasets from UOX–5-PMUA crystals at both 100 K and RT on the EMBL beamline P14 at DESY, Hamburg, Germany. This beamline provides a top-hat X-ray beam whose dimensions can be tuned to match even very large crystals (with a largest dimension often in the 0.5–1 mm range for UOX crystals). Fully bathing the entire crystal in a top-hat beam not only maximizes diffraction but, crucially, ensures a homogeneous dose distribution throughout the crystal, thereby eliminating confounding beam-shape effects.

Here, we show that the radiolysis of 5-PMUA exhibits a kinetic transition from flux-limited zero-order decay at RT to retarded target-limited first-order decay at 100 K. Kinetic modelling is consistent with a regeneration mechanism, where the organic substrate recombines with trapped O_2_ 22 times faster than it undergoes irreversible loss.

## 2. Materials and methods

### 2.1. Expression and purification of *Aspergillus flavus* UOX

*A. flavus* urate oxidase (hereafter UOX) cDNA codon-optimized for bacterial expression was purchased from GenScript (Piscataway, New Jersey, USA) and cloned using the NdeI/XhoI restriction sites in a pET-24b(+) expression vector (Novagen). Protein expression was performed as described previously (Bui *et al.*, 2014 ▸). Briefly, *Escherichia coli* BL21(DE3) cells were induced at an OD_600_ of ∼0.6–0.8 with 0.2 m*M* IPTG at 20°C for ∼20 h. Pelleted cells were resuspended in 50 m*M* Tris–HCl pH 8.0, 250 m*M* NaCl supplemented with lysozyme, DNAse and a protease-inhibitor cocktail and lysed by sonication. Protein purification was performed using a combination of ammonium sulfate precipitation, DEAE and Resource Q ion-exchange, Phenyl Sepharose hydrophobic interaction and Superdex 75 size-exclusion chromatographic steps. Fractions were analysed by SDS–PAGE and the purest fractions were pooled for further work. During the size-exclusion purification step NaCl was replaced by 30 m*M* sodium acetate in the buffer.

### 2.2. Crystallization

UOX was exchanged into Tris–acetate buffer (50 m*M* Tris pH 8.0, 10 m*M* sodium acetate) and concentrated to 20 mg ml^−1^. UOX crystals in complex with MUA were obtained at RT under anaerobic conditions using N_2_-purged UOX solutions saturated with MUA in 50 m*M* Tris–acetate buffer pH 8.0, 8% PEG 8000 as the crystallizing agent. Crystallization was carried out using the batch method by mixing reservoir and protein solutions in a 2:1 ratio. All oxygen-free crystal manipulations were carried out in an anaerobic chamber (Belle Technology) equipped with a stereomicroscope and maintained at an O_2_ concentration below 2 p.p.m.. UOX–MUA crystals belonging to space group *I*222 typically reached their maximum size within a week, and for the experiments described here the crystal dimensions ranged from 380 to 750 µm. *In crystallo* formation of the UOX–5-PMUA peroxide complex was obtained by transferring anaerobic UOX–MUA crystals to normoxic conditions (*i.e.* outside the glove box). For data collection at 100 K, crystals were exposed to air for over an hour before being transferred with a cryoloop into reservoir solution supplemented with 20% MPD, followed by rapid quenching in liquid nitrogen. For data collection at RT, a single crystal was mounted in a quartz capillary in the presence of aerobic mother liquor and sealed with wax. Excess liquid around the crystal was removed using a narrow strip of blotting paper to prevent slippage during data collection.

### 2.3. X-ray data collections

X-ray dose series were collected at 100 K and at RT from two separate UOX–5-PMUA crystals. All measurements were carried out on the P14 beamline operated by EMBL Hamburg at the PETRA III storage ring, DESY, Hamburg, Germany. The size of the top-hat X-ray beam was adjusted to exceed the crystal dimensions so that the whole crystal volume was always bathed by the beam during the experiment.

For the 100 K experiment, a UOX–5-PMUA crystal with dimensions of 580 × 470 × 420 µm was used to collect a total of 20 batches of 1440 continuous X-ray images, each corresponding to a 1° rotation. The dimensions of the X-ray beam were set at 675 µm (horizontal) × 513 µm (vertical), ensuring homogeneous illumination throughout the crystal volume. After each batch (1440° rotation), the goniometer was reset to its original position for the start of the next batch. For the RT experiment, a UOX–5-PMUA crystal with dimensions of 750 × 650 × 550 µm mounted in a glass capillary was used to collect a dose series of 35 datasets each corresponding to a 360° single-axis rotation. The top-hat beam was sized at 902 (horizontal) × 748 (vertical) µm so that, as for the 100 K experiment, it always fully bathed the crystal, ensuring homogeneous dose deposition. The beam was attenuated to achieve a flux of 1.72 × 10^12^ and 3.65 × 10^12^ photons s^−1^ for the 100 K and RT measurements, respectively.

### 2.4. Data processing

All datasets were processed using the *xia*2 package with the pipeline=3dii option, which employs *XDS* and *XSCALE* for integration and scaling, respectively (Kabsch, 2010 ▸; Winter, 2010 ▸).

For the 100 K experiment, each 1440° batch was divided into four consecutive 360° datasets, resulting in a total of 80 possible datasets. Of these, we fully analysed 14 datasets (DC1, DC3, DC5, DC7, DC9, DC13, DC17, DC21, DC28, DC35, DC45, DC56, DC69 and DC80) which sample the entire X-ray dose range of the experiment (2.34–372.36 kGy; see Section 2.5). All datasets were processed to the common maximum resolution of 1.2 Å with virtually identical statistics. Data-collection parameters and data-processing metrics are provided in Supplementary Table S1.

In contrast to the 100 K experiment, diffraction images at RT displayed a progressive loss of diffraction, becoming visibly much weaker for the latter datasets. Therefore, we processed these data in two ways. Firstly, we employed the CC_1/2_ = 0.5 criterion to define the high-resolution cutoff. Using this criterion, the maximum resolution decreased from 1.52 Å in the first dataset (DC1, 2.35 kGy) to 2.57 Å in the final dataset (DC80, 172.4 kGy). Then, as several of the metrics used to assess global radiation damage depend on the choice of the resolution cutoff, we reprocessed the first 25 datasets to the common resolution of 1.84 Å, corresponding to the maximum resolution of the highest-dose dataset within this subset. Data-collection statistics for data processing using the CC_1/2_ = 0.5 and common resolution criteria are provided in Supplementary Tables S2 and S3, respectively.

### 2.5. Estimation of the absorbed dose and analysis of specific radiation-damage effects

X-ray dose calculations were performed with the program *RADDOSE-3D* (Bury *et al.*, 2018 ▸; Dickerson *et al.*, 2024 ▸; Zeldin *et al.*, 2013 ▸). This program allows the time- and space-resolved modelling of the absorbed dose within a crystal using knowledge of the incident beam and crystal characteristics as well as the exposure time per image and number of images. Throughout this paper we report the average diffraction-weighted dose (DWD, also called fluence weighted dose, FWD) that estimates the cumulative dose within each volume element of the crystal up to a given time, weighted by the fluence through that voxel at that instant. Considering the top-hat 2D beam profile of beamline P14, which was sized to exceed the crystal dimensions, FWD coincides in our experiment with the average dose within the exposed region (here the entire crystal), thus substantially simplifying the computational modelling of the absorbed dose.

The effects of radiation damage in electron-density maps were analysed using *RIDL* (*Radiation-Induced Density Loss*), a package for the user-independent detection and quantification of radiation-induced site-specific changes to macromolecular structures as a function of absorbed dose (Bury & Garman, 2018).

### 2.6. Crystallographic refinement and model building

Crystallographic refinement was carried out with *Servalcat* (version 0.4.126; Yamashita *et al.*, 2023 ▸) that integrates and replaces some components of the *REFMAC*5 package from the *CCP*4 suite (Agirre *et al.*, 2023 ▸; Murshudov *et al.*, 2011 ▸) as well as with *phenix.refine* (Afonine *et al.*, 2012 ▸) as distributed with the *Phenix* (1.21.2-5419) suite (Liebschner *et al.*, 2019 ▸). Model changes in real space were performed with the program *Coot* (Emsley *et al.*, 2010 ▸). Details of the refinement strategies employed are provided in the relevant parts of Section 3.

### 2.7. Kinetic analysis

Dose-dependent occupancies from crystallographic refinement were modelled using a system of ordinary differential equations (ODEs) implemented in the Python programming language. Numerical integration of the ODE system was performed using the LSODA (Livermore Solver for Ordinary Differential Equations with Automatic switching) method (stiff solver) implemented in the scipy.integrate library (Python 3.9.6), with relative and absolute tolerances set to 10^−5^ and 10^−8^, respectively. Posterior probability distributions for the rate constants and population fractions were sampled using Markov chain Monte Carlo (MCMC). We employed the Affine Invariant Ensemble Sampler via the *emcee* package (Foreman-Mackey *et al.*, 2013 ▸). The log-likelihood function assumed Gaussian uncertainties based on the standard deviations derived from multiple occupancy refinement runs. Broad, uninformative priors were used for all parameters. The MCMC analysis utilized 32 walkers running for 6000 steps, with the first 1500 steps discarded as burn-in to ensure convergence. Parameter estimates are reported as the median of the posterior distribution, with uncertainties represented by the 16th and 84th percentiles. Kinetic model selection was validated using the reduced chi-squared statistic (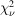) and the Bayesian Information Criterion (BIC).

## 3. Results and discussion

### 3.1. Assessment of radiation-induced global damage

Under cryogenic conditions, MX radiation-damage studies typically explore doses up to tens of MGy, resulting in global effects that include loss of diffraction, increased mosaicity, deterioration of merging statistics and an increase in Wilson *B* factors (Burmeister, 2000 ▸; de la Mora *et al.*, 2020 ▸; Leal *et al.*, 2013 ▸; Murray & Garman, 2002 ▸; Owen *et al.*, 2006 ▸; Ravelli & Garman, 2006 ▸; Krojer & von Delft, 2011 ▸; Gotthard *et al.*, 2019 ▸). Based on electron diffraction experiments at 77 K, Henderson (1990 ▸) proposed a value of ∼20 MGy as the limit at which the total diffraction intensity in MX is reduced to 50% of its initial value (*D*_0.5_). This limit was later measured at ∼40 MGy at 100 K (Owen *et al.*, 2006 ▸). In the same work, however, the authors suggested a more conservative threshold of ∼30 MGy (*D*_0.7_) as the dose above which biological interpretation may become compromised at cryogenic temperatures.

**Figure 2:**
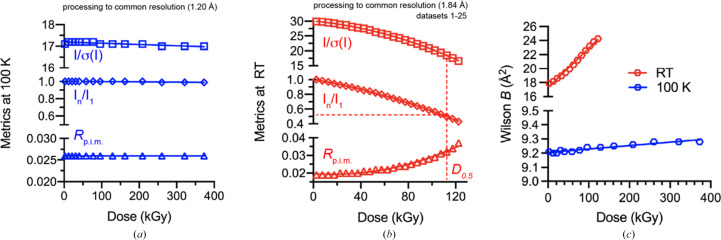
Selected dose-dependent data-processing metrics for the experiments at 100 K and RT. (*a*) Average *I*/σ(*I*), average total diffraction intensity for the *n*th dataset normalized by the average of the first (*i.e.* lowest dose) dataset and *R*_p.i.m._ for the 100 K experiment. All diffraction intensities were integrated to the common resolution of 1.2 Å. Within the dose range explored all datasets have virtually identical processing statistics. (*b*) As in (*a*) for the experiment at RT. There are clear indications of global radiation damage, with an estimated *D*_0.5_ of ∼110 kGy. (*c*) Wilson *B* values. For the RT data, *B* values are shown every other point for visual clarity.

In the present work, our primary interest was to examine the dose-dependent decay of the 5-PMUA adduct, a process that occurs at much lower doses (Bui *et al.*, 2014 ▸). Consequently, the maximum dose used in our 100 K experiment (∼0.37 MGy) was nearly two orders of magnitude lower than the ‘Henderson/Garman limits’. As expected, the crystal retained its ability to diffract to atomic resolution (all data were processed at the maximum resolution of 1.2 Å) without appreciable changes in *R*_p.i.m_, 〈*I*/σ(*I*)〉 or total diffraction intensity (Fig. 2 ▸*a* and Supplementary Table S1).

By contrast, global damage at RT progressed much faster; using a common resolution cutoff of 1.84 Å, we estimated a *D*_0.5_ value of ∼110 kGy (Fig. 2 ▸*b*). We note, however, that noticeable deterioration in data quality occurs here beyond *D*_0.5_, and up to ∼150 kGy the processing statistics remain excellent by common standards (Supplementary Table S2).

Our *D*_0.5_ value falls within the range reported by Leal *et al.* (2013 ▸) in their RT analysis of 15 different types of protein crystals but lies on the side of the most radiation-sensitive crystals, which are typically characterized by a much higher solvent content. Leal *et al.* (2013 ▸) measured *D*_0.5_ values of 0.07, 0.08 and 0.18 MGy for *P*432 crystals of *Arthrobacter nicotinovorans* 6-hydroxy-l-nicotine oxidase (solvent content 69.7%), *P*6_1_22 crystals of La Crosse orthobunyavirus L-protein polymerase N-terminal domain (solvent content 69.9%) and *I*2_1_3 crystals of bovine insulin (solvent content 67%), respectively, whereas *I*222 UOX crystals have a solvent content of ∼50%. It is possible that the high radiolytic susceptibility of 5-PMUA located at the crystallographic interface promotes radical/solvated electron generation and thereby enhances the radiation-sensitivity of UOX–5-PMUA crystals, despite their average solvent content.

Wilson *B* values have been shown to have a linear dependence on dose (*B* = *B*_0_ + β*D*), with β typically in the range 0.5–1.2 Å^2^ MGy^−1^ at cryogenic temperatures, while at RT they are higher by one to two orders of magnitude and vary widely between structures (Kmetko *et al.*, 2006 ▸; Leal *et al.*, 2011 ▸, 2013 ▸). Linear fitting of our data yielded β values of 0.24 ± 0.02 and 57 ± 1 Å^2^ MGy^−1^ at 100 K and RT, respectively (Fig. 2 ▸*c*). Although the marginally low β determined at 100 K might reflect a genuine property of these crystals, it is also possible that the narrow dose range sampled simply did not allow the full manifestation of dose-dependent *B*-value inflation, resulting in an apparent low β coefficient.

Taken together, we conclude that the 100 K experiment is essentially free of global radiation damage up to the maximum explored dose of ∼370 kGy, whereas the RT experiment displays clear hallmarks of global damage although high-quality data are obtained beyond *D*_0.5_, up to ∼150 kGy.

### 3.2. Differential lattice response at 100 K and RT

Unit-cell expansion often accompanies radiation damage in MX (Ravelli & McSweeney, 2000 ▸), but the magnitude of this effect is strongly sample-dependent (Ravelli *et al.*, 2002 ▸; Murray & Garman, 2002 ▸). The complex nature of lattice responses to radiation damage has also been discussed recently in small-molecule crystallography, highlighting the contribution of both thermal and radiation-induced expansivity components (McMonagle *et al.*, 2024 ▸).

**Figure 3:**
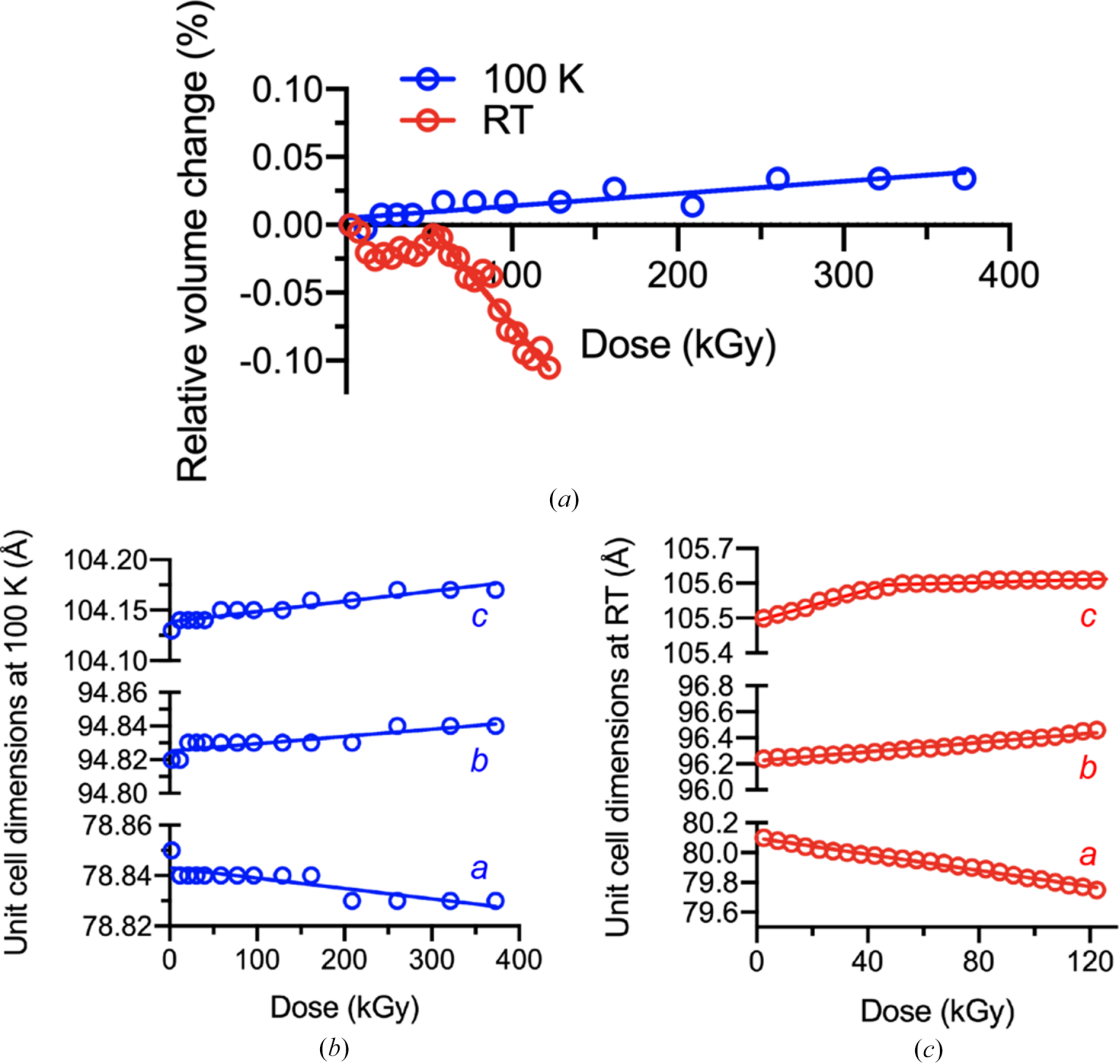
Dose-dependent changes in the UOX–5-PMUA unit cell for the experiments at 100 K and RT. (*a*) Percentage change in unit-cell volume normalized to the first dataset. (*b*) Refined unit-cell dimensions for the 100 K experiment. (*c*) Refined unit-cell dimensions for the experiment at RT. In (*b*) and (*c*) only cell axes are reported as the UOX–5-PMUA crystals belong to space group *I*222.

In our 100 K experiment, we observed a unit-cell volume increase of only 0.034% at 373 kGy relative to the first dataset (Fig. 3 ▸*a*). This expansion is marginal compared, for example, with the ∼1% increase reported for ferritin at 100 K for an absorbed dose of 2.75 MGy (Ravelli *et al.*, 2002 ▸). Cell-volume increases of between 0.5% and 1.2% have been measured for different crystals of N9 neuraminidase (Murray & Garman, 2002 ▸). The minimal increase observed in the present experiment is consistent with the virtual absence of global damage. Notably, however, the modest volume increase does not arise from uniform axis elongation: slight lengthening of the *b* and *c* axes is offset by contraction of the *a* axis, resulting in an overall nearly constant unit-cell volume across the explored dose range (Fig. 3 ▸*b*).

The behaviour at RT is more complex (Fig. 3 ▸*c*). As observed at 100 K, the *a* axis contracts with increasing dose, whereas the *b* axis expands. The *c* axis, however, initially lengthens up to ∼40 kGy and then remains essentially constant. Consequently, the unit-cell volume at RT displays a biphasic behaviour: it is essentially unchanged up to the ‘*c*-axis break point’, after which it contracts by ∼0.1% at *D*_0.5_ (Fig. 3 ▸*a*). Although we do not have explicit error estimates for the unit-cell parameters, these are expected to be the same across datasets given the identical experimental conditions. Moreover, the high density of data points defines the dose-dependent trends accurately.

As will be discussed later (Section 3.4), the complete decay of 5-PMUA at RT occurs within the first ∼40 kGy. Considering that in *I*222 UOX crystals the 5-PMUA binding site lies at a crystallographic dimer interface, it is plausible that the initial lattice behaviour reflects local structural changes arising from specific damage, whereas the subsequent volume contraction reflects the net effect of gas release. Evolution of molecular hydrogen has been proposed as the probable origin of global radiation damage at cryogenic temperatures, where limited mobility allows H_2_ to accumulate within the lattice, decreasing crystalline order and increasing unit-cell volume (Meents *et al.*, 2010 ▸). At RT, by contrast, unit-cell shrinkage has been reported to exhibit a dose-rate dependence for thaumatin and insulin crystals, being more pronounced at lower dose rates within the examined range (∼1.32–8.42 kGy s^−1^; Rajendran *et al.*, 2011 ▸). In the case of UOX–5-PMUA we propose that up to ∼40 kGy the dominant radiation-damage process is 5-PMUA radiolysis, with concomitant evolution of O_2_ causing local perturbations at the active site between symmetry-related molecules. Beyond this dose, mass loss due to H_2_ diffusion out of the crystal lattice becomes the main effect, resulting in unit-cell shrinkage likely amplified by the low dose rate employed in our RT experiment (0.92 kGy s^−1^).

### 3.3. Specific damage to the 5-PMUA adduct

**Figure 4:**
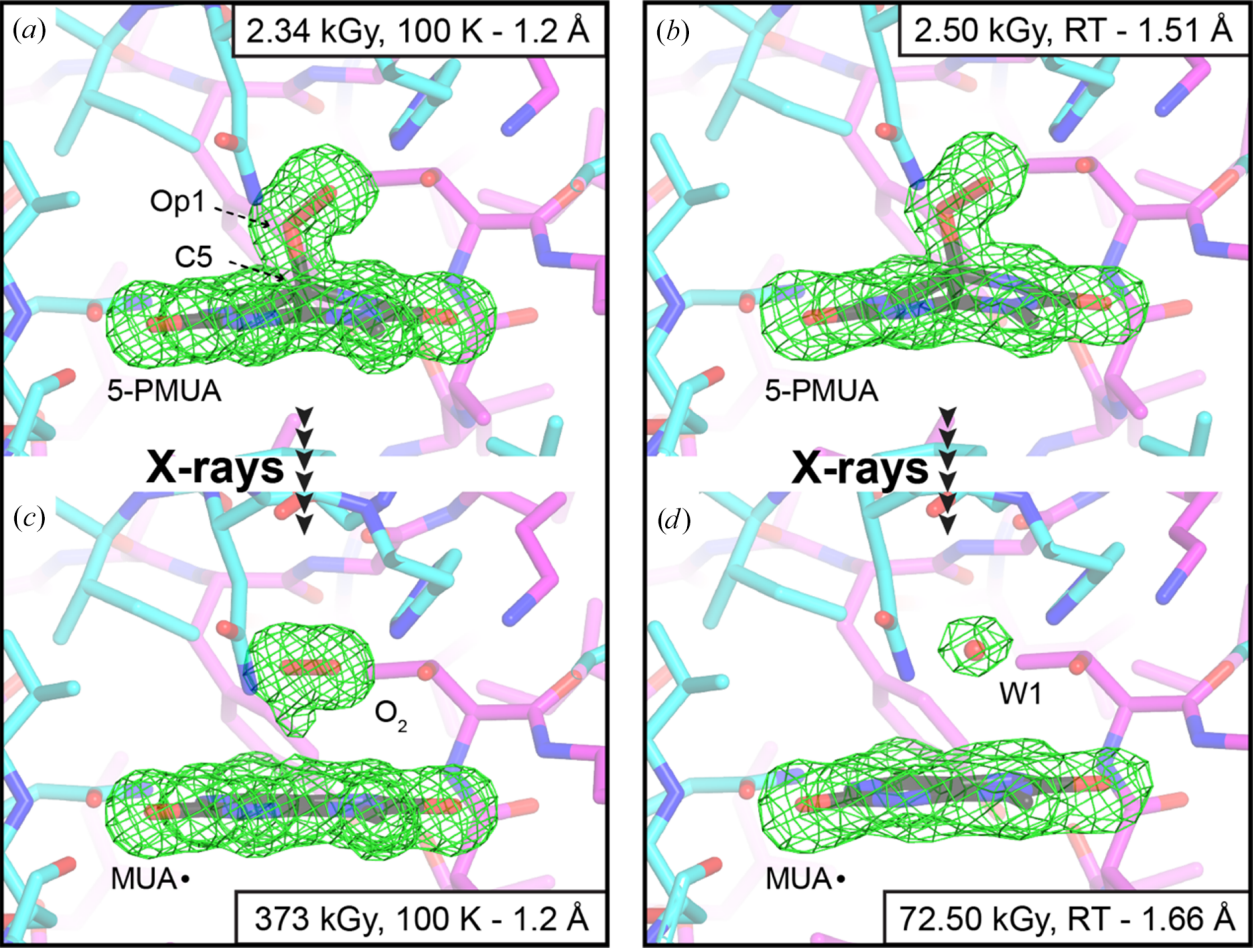
Snapshots of the UOX active site with its bound ligands at different dose points for the 100 K and RT experiments. The UOX active site is contributed by two subunits, shown here as stick representations in cyan and magenta. In all panels the *mF*_o_ − *DF*_c_ electron-density map is shown around the ligands as a green mesh displayed at the +3σ level. Ligands are shown for reference. (*a*) The 5-PMUA adduct is largely intact at 100 K and low X-ray dose (2.34 kGy). (*b*) A similar view at RT and low dose (2.50 kGy), showing the 5-PMUA species. (*c*) At high X-ray dose (373 kGy) at 100 K, the C5–Op1 bond is incompletely cleaved, with dioxygen (O_2_) trapped in the active site due to the ‘cage effect’ at cryogenic temperatures, which is critical for its partial regeneration. (*d*) At RT (72.50 kGy), 5-PMUA is fully broken; O_2_ has escaped and is replaced by a water molecule (W1) located above the MUA radical.

Preliminary inspection of difference Fourier maps clearly showed the presence of 5-PMUA with high occupancy in the lowest dose datasets of both the 100 K and RT experiments (Figs. 4 ▸*a* and 4 ▸*b*). We also calculated difference maps for the highest dose cryogenic dataset (∼373 kGy; Fig. 4 ▸*c*) and for the ∼72 kGy RT dataset (corresponding approximately to *D*_0.7_; Fig. 4 ▸*d*). At ∼72 kGy, the highest resolution is reduced by only 0.15 Å compared with the lowest dose dataset (from 1.51 to 1.66 Å) using the CC_1/2_ criterion (Supplementary Table S2).

At 100 K and maximum dose, we observed significant, yet incomplete, breakage of the C5—OO(H) bond, fully consistent with our previous observations (Bui *et al.*, 2014 ▸). Rupture of this bond is accompanied by loss of the *sp*^3^ pyramidalization at C5, yielding a planar organic structure. Concomitantly, O_2_is released and becomes trapped above this planar structure (Fig. 4 ▸*c*). As discussed in Section 1, the organic species produced by radiolysis is most likely the resonance-stabilized MUA radical (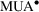), which, by analogy with the UA radical (Simic & Jovanovic, 1989 ▸), is unreactive towards ground-state O_2_.

Unlike the 100 K experiment, O_2_ does not accumulate in the ‘peroxo hole’ following complete 5-PMUA radiolysis at RT, which occurs at a much lower dose (Fig. 4 ▸*d*). Difference density maps support the idea that O_2_ leaves the active site to be replaced by a water molecule (W1 in Fig. 4 ▸*d*). A water molecule is also observed at this position in UOX–MUA or UOX–UA anaerobic complexes (Bui *et al.*, 2014 ▸; Gabison *et al.*, 2011 ▸), or in complexes with different inhibitors with a purine-type structure (Gabison *et al.*, 2010 ▸; McGregor *et al.*, 2021 ▸).

### 3.4. 5-PMUA dose-dependent decay at RT

Crystallographic refinement against the lowest dose dataset (DC1 = 2.50 kGy) at 1.51 Å resolution provided a model characterized by maps of excellent quality and statistics (*R* = 15.7% and *R*_free_ = 17.7%). UOX residues (1–295) are very well defined and only the last six C-terminal residues are too disordered to be modelled. Occupancy (*q*) refinement for 5-PMUA converged to 1 and 0.94 using *Servalcat* and *phenix.refine*, respectively. However, residual positive difference electron density in the 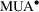 plane, as well as negative density for the C5—OO(H) bond (Supplementary Fig. S1), suggested that both 5-PMUA and 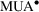 should be considered for refinement from the onset. This is reasonable as 5-PMUA breaks down naturally over several days and the crystal used for this experiment was three days old because of the time elapsed from leaving the home laboratory until beamtime. Considering that in the presence of a purine-type ligand the W1 water molecule fills the ‘peroxo-hole’ (Bui *et al.*, 2014 ▸; Gabison *et al.*, 2010 ▸, 2011 ▸; McGregor *et al.*, 2021 ▸), for occupancy refinement we applied the constraints *q*(5-PMUA) = *q*_1_, *q*(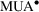) = *q*(W1) = *q*_2_ and *q*_1_*+ q*_2_ = 1. This approach converged to *q*_1_ = 0.66 and 0.72 and *q*_2_ = 0.34 and 0.28 using *Servalcat* and *phenix.refine*, respectively, with no residual difference density and *B* values consistent with the surrounding atoms.

Next, we performed independent crystallographic refinements with *Servalcat* against all datasets up to *D*_0.7_ ≃ 72 kGy (DC1–DC15) using a consistent protocol that consisted of 150 cycles of positional + isotropic ADP + occupancy refinement with ADPs initialized at the average Wilson *B* value (18 Å^2^) for all atoms. We employed harmonic restraints to stabilize the positions of 5-PMUA, 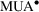 and W1 during refinement to prevent drifting at low occupancies. For each dataset, the above protocol was repeated five times using different starting *q*_1_/*q*_2_ values: 0.90/0.10, 0.75/0.25, 0.50/0.50, 0.25/0.75 and 0.10/0.90. The final *q*_1_/*q*_2_ values for each round of refinement are shown in Supplementary Table S4. For cross-validation, we also performed a single refinement run with *phenix.refine* using the same constraints above starting from randomized initial 5-PMUA, 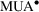 and W1 occupancies (Supplementary Table S5).

**Figure 5:**
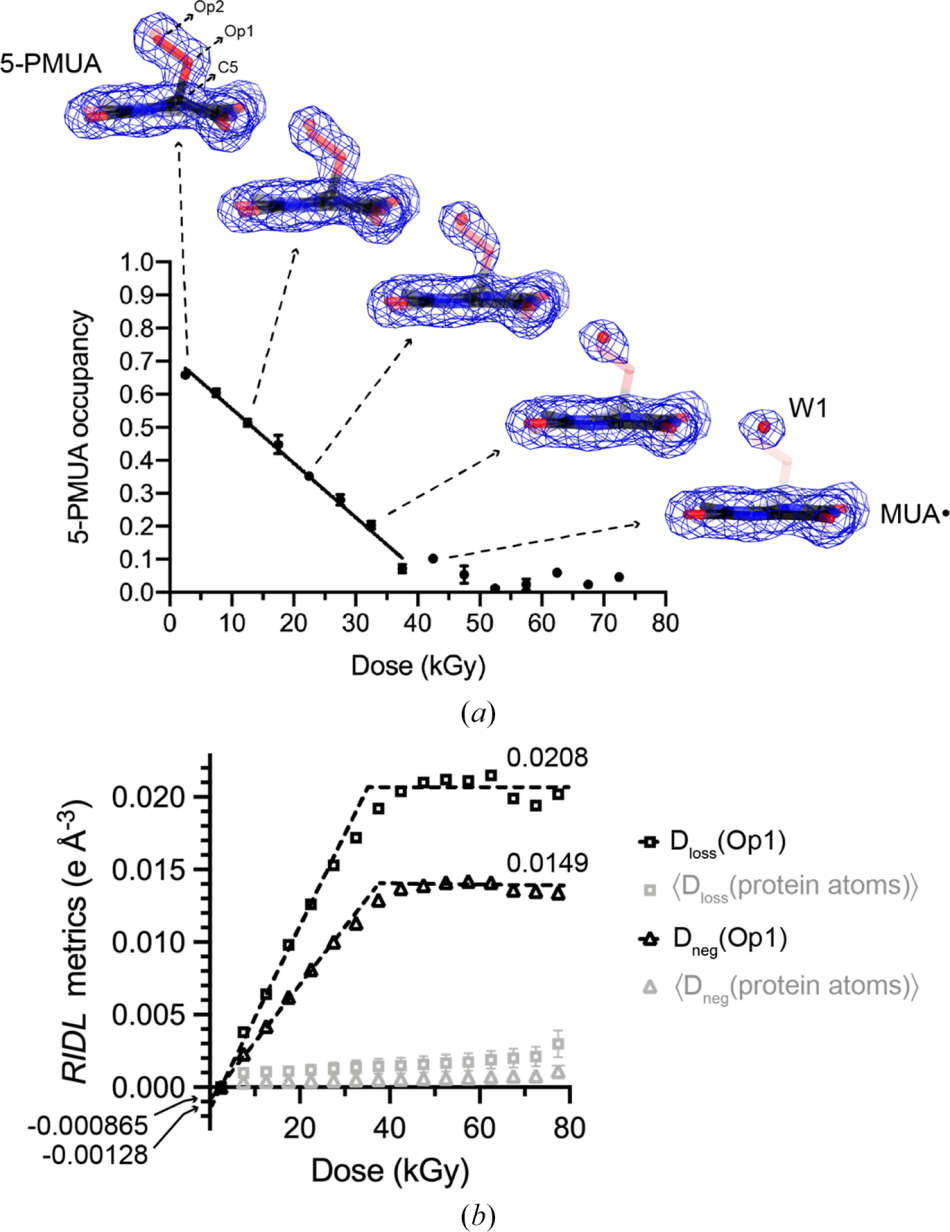
Dose-dependent 5-PMUA decay at RT. (*a*) The occupancy (*q*) of 5-PMUA derived from *Servalcat* refinement decreases linearly with dose. The values reported are the average of five independent refinement runs initialized at different occupancy values. Error bars are standard deviations. Occupancy values for MUA_T_ and W1 (not shown on the plot) correspond to 1 − *q*(5-PMUA). For selected dose points, 2*mF*_o_ − *DF*_c_ electron-density maps are shown around the bound ligands at the 1σ level. 5-PMUA, 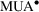 and W1 are displayed as transparent sticks, with transparency scaled to their refined occupancies (greater transparency indicates lower occupancy). As 5-PMUA radiolysis progresses, a water molecule (W1) occupies the ‘peroxo-hole’. (*b*) D_loss_ and D_neg_ values from *RIDL* for the 5-PMUA^Op1^ atom, with the average over all protein atoms shown as a reference. D_loss_(Op1) and D_neg_(Op1) plateau at about 40 kGy when 5-PMUA is fully converted to 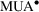. The lines represent data fit to the two regions.

The dose-dependent decay of 5-PMUA at RT is shown in Fig. 5 ▸(*a*). Linear fitting of the *Servalcat*-derived occupancies of excellent quality resulted in a decay coefficient of −0.016 kGy^−1^ (Fig. 5 ▸*a*). A nearly identical slope is obtained from *phenix.refine* values (−0.015 kGy^−1^; Supplementary Fig. S2). These results indicate that the 5-PMUA present at *D* = 0, which extrapolates to *q*_1_ = 0.72 (*Servalcat*) or 0.77 (*phenix.refine*), is virtually fully radiolysed within 40–50 kGy.

We further analysed the dose-dependent decay of 5-PMUA using *RIDL* (Bury & Garman, 2018 ▸). A useful metric provided by this program to monitor radiation damage at the atomic level is D_loss_(atom).^1^ This quantity provides a measure of the maximum density loss and is the per-atom equivalent of the magnitude of negative Fourier difference map peaks in units of electrons per Å^3^. D_loss_(atom) has been previously employed to analyse the differential radiation-damage susceptibility in a protein–RNA complex (Bury *et al.*, 2016 ▸) and in a survey of radiation-induced changes to tyrosine residues in MX (Bury *et al.*, 2017 ▸). Another meaningful quantity calculated within *RIDL* is D_neg_(atom) that considers the contribution of all electron-density-weighted voxels that belong to an atom. As shown in Fig. 5 ▸(*b*), both D_loss_(Op1) and D_neg_(Op1) are linear with dose up to ∼40 kGy and increase at a rate which is substantially faster than the average for all protein atoms, which reflects global damage. From the linear fitting of D_loss_(Op1) and D_neg_(Op1) we calculated rates of 5-PMUA decay of 2.2% kGy^−1^ and 2.3% kGy^−1^, respectively, that are in excellent agreement with the value of 1.6% kGy^−1^ estimated from occupancy refinement.

Interestingly, in a previous RT serial synchrotron crystallography (SSX) experiment, we observed no significant radiation damage to 5-PMUA for doses of the order of tens of kilograys, with an estimated upper limit of around 70 kGy (Zielinski *et al.*, 2022 ▸). We believe that this discrepancy can arise from fundamental methodological differences as well as dose-rate regimes that differ by orders of magnitude. A detailed analysis of how these variables influence radiation damage is the focus of ongoing work, for which the UOX–5-PMUA complex serves as an ideal model system.

### 3.5. 5-PMUA dose-dependent decay at 100 K

We initially tested the hypothesis that MUA had completely reacted with O_2_ to produce 5-PMUA and that no radiolysis had taken place at the lowest dose (2.34 kGy). However, following crystallographic refinement the presence of minor positive difference density at the position where O_2_ is expected (Supplementary Fig. S3*a*), which was more significant for the subsequent dose point at 11.7 kGy (Supplementary Fig. S3*b*), suggested that even the early dose points are not totally free of radiation damage.

Occupancy refinement against the lowest dose dataset using the constraint *q*_1_*+ q*_2_ = 1. where *q*(5-PMUA) = *q*_1_ and *q*(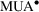) = *q*(O_2_) = *q*_2_, converged to *q*(5-PMUA) = 0.836. We deemed a 16.4% 5-PMUA decay within 2.34 kGy at 100 K to be very unlikely given that we estimated this to be in the range 4–5.75% at RT. This prompted us to explore a more general hypothesis that assumed the additional presence of unreacted MUA at the start of the experiment (*D* = 0). Taking into account that 5-PMUA was produced *in crystallo* by allowing O_2_ (air) to diffuse for 1 h within a ∼30 µl anaerobic PEG 8000-based crystallization drop and that reactions in the crystal state are significantly slower compared with the solution state, this scenario appears to be likely (Makinen & Fink, 1977 ▸; Mozzarelli & Rossi, 1996 ▸; Tosha *et al.*, 2017 ▸).

The exact chemical identity of UOX-bound UA that is activated for O_2_ attack has not yet been firmly established. An early study proposed that a Lys–Thr catalytic dyad deprotonates the bound UA monoanion to generate the reactive UA dianion (Imhoff *et al.*, 2003 ▸). This has been challenged by a more recent theoretical study that proposed a PCET mechanism mediated by the Lys–Thr dyad but without formal deprotonation of the UA monoanion (Wei *et al.*, 2017 ▸). Neutron diffraction studies on chloride-inhibited UOX–UA have shown that the substrate binds as the iminol tautomer (as shown in Fig. 1 ▸*a*; Oksanen *et al.*, 2014 ▸); however, it is unclear whether chloride prevents subsequent deprotonation and activation or whether the observed species is the reactive form.

Like UA, the exact chemical identity of UOX-activated MUA is unknown, although the same activation mechanism is likely to apply. Even if chemically distinct, MUA (monoanion or dianion) and 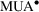 will be structurally indistinguishable by X-ray diffraction, particularly if present as a mixture. Thus, for the purpose of crystallographic refinement, MUA and 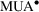 can be grouped into a single species that we refer to as MUA_T_ (Total). Using the occupancy constraints *q*(5-PMUA) + *q*(MUA_T_) = 1, *q*(5-PMUA) + *q*(O_2_) + *q*(W1) = 1 and *q*(MUA_T_) = *q*(O_2_) + *q*(W1), we performed five independent refinement jobs for the 2.34 kGy dataset with *Servalcat* starting from different occupancy values that converged to *q*(5-PMUA) = 0.81 ± 0.02, *q*(MUA_T_) = 0.19 ± 0.05, *q*(O_2_) = 0.097 ± 0.006 and *q*(W1) = 0.09 ± 0.02. As O_2_ is generated from 5-PMUA rupture, we can infer that ∼10% of 5-PMUA breakage occurs within the initial 2.34 kGy. This is closer but still marginally higher than what we estimated for a similar dose at RT. A possible explanation for this is that whilst the absorbed dose at RT contributes to both general and 5-PMUA specific damage, the same dose at 100 K leads only to 5-PMUA rupture, acting solely on the most radiation-sensitive bond in the structure. This agrees with the observation that specific and global radiation damage are more coupled at RT than they are at cryogenic temperatures (Gotthard *et al.*, 2019 ▸).

**Figure 6:**
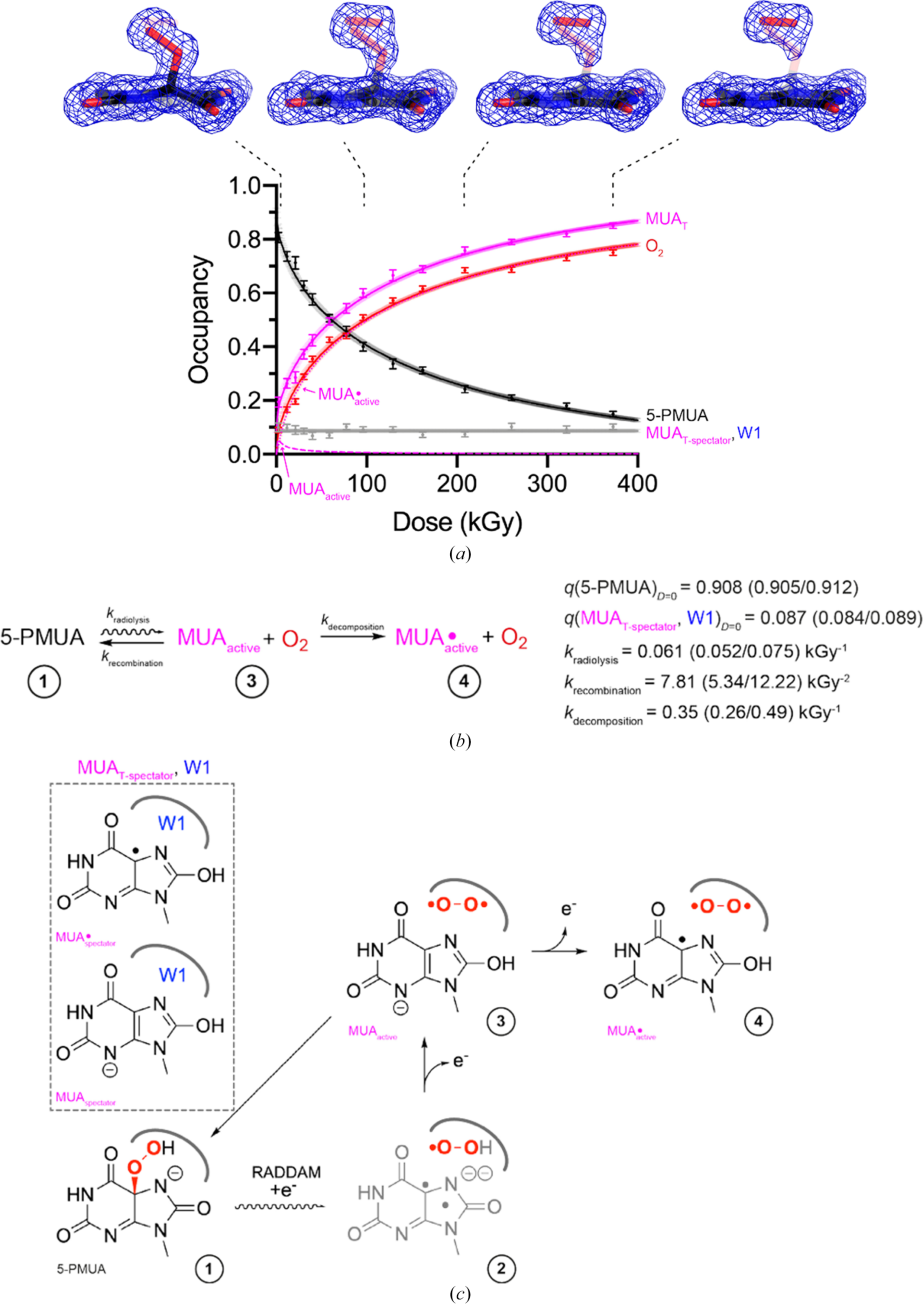
Dose-dependent 5-PMUA decay at 100 K. (*a*) Occupancy values from *Servalcat* refinement for the species indicated in the legend are shown as coloured circles with error bars corresponding to their standard deviations calculated from multiple refinement rounds as indicated in the main text. The curves represent the kinetic fit according to the scheme shown in (*b*). Transparent error bars are lower and upper confidence intervals. For selected dose points, 2*mF*_o_ − *DF*_c_ electron-density maps are shown around the bound ligands at the 1σ level. 5-PMUA, MUA_T_ and O_2_ are displayed as transparent sticks, with transparency scaled to their refined occupancies (greater transparency indicates lower occupancy). W1, although present, is essentially invisible because of its low occupancy. (*b*) Kinetic scheme employed to model the experimental occupancies. Fitted parameters with confidence intervals are reported. (*c*) Proposed molecular mechanism for 5-PMUA decomposition and regeneration.

A consistent refinement protocol was then applied to all datasets of the dose series. Starting from the fully refined lowest dose model, we initialized all ADPs to the Wilson *B* value (9.25 Å^2^) and performed (i) 150 cycles of positional + isotropic ADP + occupancy refinement followed by (ii) 50 cycles of positional + anisotropic ADP + occupancy refinement with *Servalcat*. Occupancies were refined for 5-PMUA, MUA_T_, O_2_ and W1 using the constraints defined above. For every dose point we carried out five independent refinement rounds each starting from a different combination of occupancy values. Harmonic restraints were applied to 5-PMUA, MUA_T_, O_2_ and W1 coordinates to prevent drifting when their occupancy is low. H atoms were included at riding positions. Final occupancies for all runs are given in Supplementary Table S6. Average occupancies are given in Fig. 6 ▸(*a*) together with their kinetic modelling according to the reaction scheme shown in Fig. 6 ▸(*b*).

### 3.6. Kinetic analysis

We tested various possible kinetic models, and that in Fig. 6 ▸(*b*) fits the experimental data best (Fig. 6 ▸*a*). It assumes a 5-PMUA regeneration step from the MUA–O_2_ pair that is produced in the active site following its radiolysis. It is important to note that only MUA generated from 5-PMUA radiolysis will contribute to its recovery as it coexists with O_2_ in the ‘peroxo-hole’. We therefore define MUA and 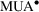 resulting from 5-PMUA radiolysis as ‘active’ (MUA_T-active_ = MUA_active_ + 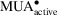). The occupancy profile of MUA_active_ and 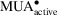 in the low-dose region is shown in Supplementary Fig. S4. Our model also allows the existence of a fraction of MUA and 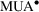 (MUA_T-spectator_ = MUA_spectator_ + 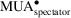) present prior to cryocooling. This fraction which is coupled with W1 in the ‘peroxo-hole’ is a mere spectator and does not intervene in the reaction. In agreement with this, occupancy refinement shows this fraction to be dose-independent.

The ODEs system of five variables, *k*_radiolysis_, *k*_recombination_, *k*_decomposition_, *q*(5-PMUA)_*D*=0_ and *q*(MUA_T-spectator_)_*D*=0_ = *q*(W1)_*D*=0_, was solved using the LSODA method. Posterior probability distributions for the rate constants and population fractions were sampled using the *MCMC* algorithm. The system is well behaved numerically and we obtained [median (lower CI/upper CI)]: *k*_radiolysis_ = 0.061 (0.052/0.075) kGy^−1^, *k*_recombination_ = 7.806 (5.342/12.216) kGy^−2^, *k*_decomposition_ = 0.347 (0.262/0.494) kGy^−1^, *q*(5-PMUA)_*D*=0_ = 0.908 (0.905/0.912) and *q*(W1)_*D*=0_ = *q*(MUA_T-spectator_)_*D*=0_ = 0.087 (0.084/0.089) kGy^−1^. Remarkably, although the sum of *q*(5-PMUA)_*D*=0_ and *q*(MUA_T-spectator_)_*D*=0_ = *q*(W1)_*D*=0_ was left unconstrained the sum of their medians is 0.995, which is a near-perfect mass balance.

The kinetic competition within the active site is quantified by the branching ratio *k*_recombination_/*k*_decomposition_ ≃ 22.2, which reveals an efficient self-healing mechanism. Once the MUA–O_2_ pair is formed, it is over 20 times more likely to recombine to regenerate 5-PMUA than to undergo irreversible degradation to 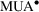. This dominance of recombination over damage explains the marked retardation of radiolysis at higher doses. Furthermore, the low forward equilibrium constant *k*_radiolysis_/*k*_recombination_ ≃ 0.008 indicates that balance strongly favours the intact 5-PMUA molecule, ensuring that the transient MUA_active_–O_2_ intermediate does not accumulate significantly but rather serves as a short-lived gateway for the repair cycle.

As previous theoretical calculations suggested that one-electron reduction, unlike one-electron oxidation, leads to an unstable radical resulting in C5—OO(H) bond breakage (Bui *et al.*, 2014 ▸), we therefore propose the scheme in Fig. 6 ▸(*c*) for the overall radiolysis/recombination mechanism. This scheme involves the transient species **2** that does not accumulate, resulting in *k*_radiolysis_ as the effective rate constant for the transition from **1** to **3**.

Alternative kinetic models were evaluated to test the robustness of our recombination model. A simple first-order decay model failed to capture the occupancy profiles (ΔBIC > 1300, 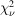 = 26.6), demonstrating that the radiolytic process is not a simple irreversible dissociation (Supplementary Fig. S5). We further compared our model against an empirical power-law model, rate ∝ *q*(5-PMUA)^*n*^. Although the power law (*n* ≃ 2.2) mimicked the dispersive slowing of the decay, the recombination model yielded a significantly lower BIC score (ΔBIC ≃ 45). This provides strong statistical evidence that the reaction is driven by a specific product-dependent recombination pathway involving a transient intermediate, rather than generic kinetic dispersion.

### 3.7. Target-limited versus flux-limited regime

A comparison of the 5-PMUA decay profiles at RT and 100 K reveals that irrespective of the MUA–O_2_ recombination that occurs at cryogenic temperature, the radiolytic process is fundamentally different. Whilst 5-PMUA decay at RT follows zero-order kinetics, implying a reaction rate that is independent of substrate concentration (occupancy), cryogenic data require its modelling as a first-order reaction, d*q*(5-PMUA)/d*D* = *k*_radiolysis_*q*(5-PMUA), that depends on its occupancy. This shift indicates a fundamental change in the energy-transfer dynamics. We interpreted this in terms of the different mobility of the radiolytic species. At higher temperatures, thermal energy allows free radicals to diffuse significantly through the crystal lattice, thus establishing a ‘flux-limited’ regime: essentially all mobile reactive species generated by the constant X-ray flux succeed in finding and reacting with a 5-PMUA target, regardless of its instantaneous concentration. Consequently, the global reaction rate becomes locked to the steady rate of radical generation by the beam rather than the depletion of the substrate. Furthermore, the higher mobility at RT facilitates the diffusion of O_2_ out of the ‘peroxo-hole’ and its replacement by W1, thereby precluding the recombination pathway observed at 100 K. At cryogenic temperature, 5-PMUA decays with first-order kinetics that are characteristic of a target-limited regime. Thus, radiolysis must rely on the direct, localized deposition of energy at or near a specific 5-PMUA site; thus, the reaction probability scales linearly with the number of remaining targets.

## 4. Conclusions

This study confirms our previous findings that alongside radiolysis, a mechanism of peroxide regeneration is operational in cofactor-independent urate oxidase crystals under cryogenic conditions (Bui *et al.*, 2014 ▸). We observe that the ‘cage effect’ at 100 K traps the radiolytically cleaved dioxygen within the active site, facilitating a recombination mechanism that is kinetically inaccessible at room temperature, where the product irreversibly diffuses into the bulk solvent. This finding reinforces the critical distinction between global diffraction loss and specific chemical damage. Moreover, these data suggest that controlled radiolysis, when carefully calibrated, can be not only an experimental limitation to be minimized, but also a tool to probe redox reaction mechanisms and transient states that are otherwise elusive.

## Supplementary Material

Supplementary Tables and Figures. DOI: 10.1107/S2059798326002688/xh5067sup1.pdf

## Footnotes

1 In the original reference this quantity is referred to as *D*_loss_(atom). However, as we use an italic *D* for dose here, we have employed a roman D to indicate *RIDL* density metrics.
