# Supplementary material for: Radiation-induced peroxide rupture and its temperature-dependent repair probed by homogeneous X-ray irradiation
Source: Acta Crystallogr D Struct Biol. 2026 Apr 13;82(Pt 5):492–503. doi: 10.1107/S2059798326002688 (PMC13133987; doi:10.1107/S2059798326002688)
Supplement: Supplementary file 1 [file d-82-00492-sup1.pdf]

STRUCTURAL
BIOLOGY

Volume 82 (2026)

Supporting information for article:

Radiation-induced peroxide rupture and its temperature-dependent repair probed by homogeneous X-ray irradiation

Symeon Koulas, Soi Bui, Julius B Kirkegaard, Gleb Bourenkov and Roberto A Steiner

Supplementary Table S1 (part 1 of 2). Data collection statistics for the experiment at 100 K.

Data set	1	3	5	7	9	13	17
Beam Line	P14 (DESY)						
Beam shape	Top-hat						
Flux (photons/s)	1.72376E+12						
Exposure time per image (s)	0.0752						
Δφ per image (°)	1						
Total exposure time per dataset (s)	2.7072						
Collimation (µm)	674.62 horizontal / 512.61 vertical						
Crystal size (µm)	580 × 470 × 420						
Wavelength (Å)	0.97625						
Temperature (K)	100						
Space group	*I*222						
Cell dimensions (Å) *a b c*	78.85 94.82 104.13	78.84 94.82 104.14	78.84 94.83 104.14	78.84 94.83 104.14	78.84 94.83 104.14	78.84 94.83 104.15	78.84 94.83 104.15
Resolution range (Å) Highest res. bin (Å)	47.45-1.20 (1.22-1.20)	47.45-1.20 (1.22-1.20)	47.45-1.20 (1.22-1.20)	47.45-1.20 (1.22-1.20)	47.45-1.20 (1.22-1.20)	47.45-1.20 (1.22-1.20)	47.45-1.20 (1.22-1.20)
Unique reflections	118667 (5784)	118659 (5780)	118687 (5787)	118680 (5786)	118676 (5785)	118702 (5785)	118698 (5785)
Overall redundancy	13.5 (13.5)	13.5 (13.5)	13.5 (13.5)	13.5 (13.5)	13.5 (13.5)	13.5 (13.5)	13.5 (13.5)
Completeness, (%)	97.7 (96.0)	97.7 (96.0)	97.7 (96.0)	97.7 (96.0)	97.7 (96.0)	97.7 (96.0)	97.7 (96.0)
CC(1/2)	1.00 (0.835)	1.00 (0.838)	1.00 (0.839)	1.00 (0.844)	1.00 (0.841)	1.00 (0.837)	1.00 (0.842)
R_{pim} (I)	0.026 (0.352)	0.026 (0.351)	0.026 (0.350)	0.026 (0.357)	0.026 (0.355)	0.026 (0.354)	0.026 (0.354)
⟨I/σ(I)⟩	17.1 (2.1)	17.2 (2.1)	17.2 (2.2)	17.2 (2.1)	17.2 (2.1)	17.2 (2.1)	17.2 (2.1)
Wilson *B* value ($Å^2$)	9.21	9.20	9.20	9.22	9.21	9.21	9.22
DWD (kGy)	2.34	11.73	21.11	30.49	39.87	58.63	77.39

continues on the next page…

Supplementary Table S1 (part 2 of 2). Data collection statistics for the experiment at 100 K.

…continues from the previous page

Data set	21	28	35	45	56	69	80
Beam Line	P14 (DESY)						
Beam shape	Top-hat						
Flux (photons/s)	1.72E+12						
Exposure time per image (s)	0.0752						
Δφ per image (°)	1						
Total exposure time per dataset (s)	2.7072						
Collimation (μm)	675 horizontal / 513 vertical						
Crystal size (μm)	580 × 470 × 420						
Wavelength (Å)	0.97625						
Temperature (K)	100						
Space group	*I*222						
Cell dimensions (Å) *a b c*	78.84 94.83 104.15	78.84 94.82 104.15	78.84 94.83 104.16	78.83 94.83 104.16	78.83 94.84 104.17	78.83 94.84 104.17	78.83 94.84 104.17
Resolution range (Å) Highest res. bin (Å)	47.45-1.20 (1.22-1.20)	47.45-1.20 (1.22-1.20)	47.45-1.20 (1.22-1.20)	47.45-1.20 (1.22-1.20)	47.46-1.20 (1.22-1.20)	47.46-1.20 (1.22-1.20)	47.45-1.20 (1.22-1.20)
Unique reflections	118692 (5785)	118682 (5783)	118697 (5777)	118681 (5773)	118821 (5760)	118704 (5763)	118698 (5760)
Overall redundancy	13.5 (13.5)	13.5 (13.5)	13.5 (13.5)	13.5 (13.5)	13.4 (13.5)	13.5 (13.5)	13.5 (13.5)
Completeness, (%)	97.7 (96.0)	97.7 (96.0)	97.7 (96.0)	97.7 (96.0)	97.8 (96.0)	97.7 (96.0)	97.7 (96.0)
CC(1/2)	1.00 (0.842)	1.00 (0.840)	1.00 (0.832)	1.00 (0.835)	1.00 (0.833)	1.00 (0.833)	1.00 (0.829)
R_{pim} (I)	0.026 (0.356)	0.026 (0.356)	0.026 (0.356)	0.026 (0.358)	0.026 (0.362)	0.026 (0.364)	0.026 (0.364)
⟨I/σ(I)⟩	17.1 (2.1)	17.1 (2.1)	17.1 (2.1)	17.1 (2.1)	17.0 (2.1)	17.0 (2.1)	17.0 (2.1)
Wilson *B* value (Å^2)	9.24	9.24	9.25	9.26	9.28	9.28	9.28
DWD (kGy)	96.15	128.98	161.81	208.71	260.30	321.27	372.86

Supplementary Table S2 (part 1 of 7). Data collection and processing statistics [*CC*(1/2) = 0.5 cut-off criterion] for the experiment at RT.

Data set	1	2	3	4	5
Beam Line	P14 (DESY)				
Beam shape	Top-hat				
Flux (photons/s)	3.65E+12				
Exposure time per image (s)	0.0752				
Δφ per image (°)	1				
Total exposure time per dataset (s)	2.7072				
Collimation (μm)	902 horizontal / 748 vertical				
Crystal size (μm)	750 × 650 × 550				
Wavelength (Å)	0.97625				
Temperature (K)	293				
Space group	*I*222				
Cell dimensions (Å) *a b c*	80.10 96.23 105.49	80.07 96.24 105.50	80.05 96.25 105.52	80.03 96.25 105.53	80.02 96.26 105.54
Resolution range (Å) Highest res. bin (Å)	30.75-1.51 (1.54-1.51)	30.75-1.52 (1.55-1.52)	30.75-1.52 (1.55-1.52)	30.75-1.53 (1.56-1.53)	30.75-1.54 (1.57-1.54)
Unique reflections	63952 (3167)	62705 (3141)	62696 (3135)	61506 (3055)	60308 (2975)
Overall redundancy	13.5 (13.3)	13.5 (13.3)	13.5 (13.3)	13.5 (13.3)	13.5 (13.3)
Completeness, (%)	99.8 (99.8)	99.8 (99.8)	99.8 (99.8)	99.8 (99.8)	99.8 (100.0)
CC(1/2)	0.999 (0.526)	0.999 (0.514)	0.999 (0.508)	0.999 (0.489)	0.999 (0.487)
R_{pim} (I)	0.030 (0.744)	0.030 (0.725)	0.031 (0.771)	0.030 (0.749)	0.030 (0.759)
⟨I/σ(I)⟩	17.5 (1.2)	17.7 (1.2)	17.5 (1.1)	17.7 (1.1)	17.7 (1.1)
Wilson *B* value (Å^2)	15.36	15.59	15.68	15.85	16.14
DWD (kGy)	2.50	7.50	12.49	17.49	22.49

continues on the next page…

Supplementary Table S2 (part 2 of 7). Data collection and processing statistics [*CC*(1/2) = 0.5 cut-off criterion] for the experiment at RT.

…continues from the previous page

Data set	6	7	8	9	10
Beam Line	P14 (DESY)				
Beam shape	Top-hat				
Flux (photons/s)	3.65E+12				
Exposure time per image (s)	0.0752				
Δφ per image (°)	1				
Total exposure time per dataset (s)	2.7072				
Collimation (μm)	902 horizontal / 748 vertical				
Crystal size (μm)	750 × 650 × 550				
Wavelength (Å)	0.97625				
Temperature (K)	293				
Space group	*I*222				
Cell dimensions (Å) *a b c*	80.00 96.27 105.55	80.00 96.28 105.56	79.98 96.28 105.57	79.97 96.29 105.58	79.97 96.29 105.58
Resolution range (Å) Highest res. bin (Å)	30.75-1.55 (1.58-1.55)	30.75-1.56 (1.59-1.56)	30.75-1.57 (1.60-1.57)	30.75-1.58 (1.61-1.58)	30.75-1.59 (1.62-1.59)
Unique reflections	59166 (2946)	58055 (2877)	56931 (2790)	55881 (2770)	54868 (2705)
Overall redundancy	13.5 (13.3)	13.6 (13.3)	13.6 (13.4)	13.6 (13.4)	13.6 (13.4)
Completeness, (%)	99.8 (99.9)	99.8 (99.9)	99.7 (99.9)	99.8 (99.9)	99.7 (100.0)
CC(1/2)	0.999 (0.503)	0.999 (0.492)	0.999 (0.488)	0.999 (0.525)	0.999 (0.535)
R_{pim} (I)	0.030 (0.789)	0.030 (0.738)	0.030 (0.730)	0.030 (0.772)	0.030 (0.760)
⟨I/σ(I)⟩	17.8 (1.1)	17.8 (1.2)	17.9 (1.2)	18.0 (1.1)	17.9 (1.2)
Wilson *B* value ($Å^2$)	16.34	16.59	16.80	17.07	17.29
DWD (kGy)	27.49	32.48	37.48	42.48	47.48

continues on the next page…

Supplementary Table S2 (part 3 of 7). Data collection and processing statistics [*CC*(1/2) = 0.5 cut-off criterion] for the experiment at RT.

…continues from the previous page

Data set	11	12	13	14	15
Beam Line	P14 (DESY)				
Beam shape	Top-hat				
Flux (photons/s)	3.65E+12				
Exposure time per image (s)	0.0752				
Δφ per image (°)	1				
Total exposure time per dataset (s)	2.7072				
Collimation (μm)	902 horizontal / 748 vertical				
Crystal size (μm)	750 × 650 × 550				
Wavelength (Å)	0.97625				
Temperature (K)	293				
Space group	*I*222				
Cell dimensions (Å) *a b c*	79.96 96.31 105.59	79.95 96.31 105.59	79.94 96.32 105.59	79.92 96.33 105.60	79.91 96.34 105.60
Resolution range (Å) Highest res. bin (Å)	30.75-1.60 (1.63-1.60)	30.75-1.61 (1.64-1.61)	30.75-1.62 (1.65-1.62)	30.75-1.64 (1.67-1.64)	30.75-1.66 (1.69-1.66)
Unique reflections	53863 (2665)	52862 (2644)	51911 (2566)	50042 (2487)	48282 (2383)
Overall redundancy	13.6 (13.4)	13.6 (13.3)	13.6 (13.3)	13.6 (13.3)	13.6 (13.3)
Completeness, (%)	99.7 (100.0)	99.7 (99.9)	99.7 (99.9)	99.7 (99.8)	99.7 (99.9)
CC(1/2)	0.999 (0.535)	0.999 (0.521)	0.999 (0.470)	0.999 (0.474)	0.999 (0.475)
R_{pim} (I)	0.030 (0.760)	0.031 (0.752)	0.031 (0.776)	0.031 (0.825)	0.031 (0.796)
⟨I/σ(I)⟩	17.8 (1.2)	17.8 (1.1)	17.7 (1.1)	17.9 (1.1)	18.2 (1.1)
Wilson *B* value ($Å^2$)	17.56	17.83	18.08	18.55	19.00
DWD (kGy)	52.47	57.47	62.47	67.47	72.47

continues on the next page…

Supplementary Table S2 (part 4 of 7). Data collection and processing statistics [*CC*(1/2) = 0.5 cut-off criterion] for the experiment at RT.

…continues from the previous page

Data set	16	17	18	19	20
Beam Line	P14 (DESY)				
Beam shape	Top-hat				
Flux (photons/s)	3.65E+12				
Exposure time per image (s)	0.0752				
Δφ per image (°)	1				
Total exposure time per dataset (s)	2.7072				
Collimation (µm)	902 horizontal / 748 vertical				
Crystal size (µm)	750 × 650 × 550				
Wavelength (Å)	0.97625				
Temperature (K)	293				
Space group	*I*222				
Cell dimensions (Å) *a b c*	79.90 96.35 105.60	79.89 96.36 105.61	79.87 96.37 105.61	79.85 96.38 105.61	79.83 96.39 105.61
Resolution range (Å) Highest res. bin (Å)	30.75-1.67 (1.70-1.67)	30.75-1.68 (1.71-1.68)	30.75-1.71 (1.74-1.71)	30.75-1.72 (1.75-1.72)	30.75-1.74 (1.77-1.74)
Unique reflections	47396 (2331)	46599 (2310)	44206 (2202)	43441 (2132)	41953 (2046)
Overall redundancy	13.6 (13.3)	13.6 (13.3)	13.6 (13.4)	13.6 (13.3)	13.6 (13.2)
Completeness, (%)	99.7 (100.0)	99.7 (99.9)	99.7 (99.9)	99.7 (99.8)	99.7 (99.8)
CC(1/2)	0.999 (0.467)	0.999 (0.463)	0.999 (0.532)	0.999 (0.501)	0.999 (0.552)
R_{pim} (I)	0.031 (0.784)	0.032 (0.796)	0.031 (0.730)	0.032 (0.732)	0.032 (0.795)
⟨I/σ(I)⟩	18.0 (1.1)	17.7 (1.1)	18.1 (1.1)	17.8 (1.1)	17.7 (1.1)
Wilson *B* value ($Å^2$)	19.33	19.69	20.40	20.76	21.32
DWD (kGy)	77.46	82.46	87.46	92.46	97.46

continues on the next page…

Supplementary Table S2 (part 5 of 7). Data collection and processing statistics [$CC(1/2)$ = 0.5 cut-off criterion] for the experiment at RT.

…continues from the previous page

Data set	21	22	23	24	25
Beam Line	P14 (DESY)				
Beam shape	Top-hat				
Flux (photons/s)	3.65E+12				
Exposure time per image (s)	0.0752				
Δφ per image (°)	1				
Total exposure time per dataset (s)	2.7072				
Collimation (µm)	902 horizontal / 748 vertical				
Crystal size (µm)	750 × 650 × 550				
Wavelength (Å)	0.97625				
Temperature (K)	293				
Space group	*I*222				
Cell dimensions (Å) *a b c*	79.81 96.40 105.61	79.80 96.41 105.61	79.78 96.42 105.61	79.77 96.45 105.61	79.75 96.46 105.61
Resolution range (Å) Highest res. bin (Å)	30.75-1.75 (1.78-1.75)	30.75-1.77 (1.80-1.77)	30.75-1.79 (1.82-1.79)	30.75-1.81 (1.84-1.81)	30.75-1.84 (1.87-1.84)
Unique reflections	41261 (2051)	39896 (1959)	38571 (1902)	37325 (1808)	35576 (1756)
Overall redundancy	13.7 (13.2)	13.7 (13.2)	13.7 (13.4)	13.7 (13.4)	13.7 (13.2)
Completeness, (%)	99.7 (100.0)	99.7 (99.9)	99.7 (99.9)	99.7 (99.9)	99.7 (99.9)
$CC(1/2)$	0.999 (0.537)	0.999 (0.454)	0.999 (0.483)	0.999 (0.446)	0.999 (0.517)
R_{pim} (I)	0.033 (0.875)	0.034 (0.857)	0.035 (0.799)	0.036 (0.784)	0.037 (0.729)
⟨I/σ(I)⟩	17.3 (0.9)	17.3 (0.9)	17.0 (1.0)	16.7 (1.1)	16.5 (1.1)
Wilson *B* value (Å^2)	21.74	22.27	22.85	23.39	24.22
DWD (kGy)	102.45	107.45	112.45	117.45	122.45

continues on the next page…

Supplementary Table S2 (part 6 of 7). Data collection and processing statistics [*CC*(1/2) = 0.5 cut-off criterion] for the experiment at RT.

…continues from the previous page

Data set	26	27	28	29	30
Beam Line	P14 (DESY)				
Beam shape	Top-hat				
Flux (photons/s)	3.65E+12				
Exposure time per image (s)	0.0752				
Δφ per image (°)	1				
Total exposure time per dataset (s)	2.7072				
Collimation (μm)	902 horizontal / 748 vertical				
Crystal size (μm)	750 × 650 × 550				
Wavelength (Å)	0.97625				
Temperature (K)	293				
Space group	*I*222				
Cell dimensions (Å) *a b c*	79.72 96.47 105.60	79.71 96.49 105.61	79.69 96.50 105.60	79.66 96.50 105.58	79.67 96.53 105.59
Resolution range (Å) Highest res. bin (Å)	30.75-1.86 (1.89-1.86)	30.75-1.89 (1.92-1.89)	30.75-1.92 (1.95-1.92)	30.75-1.95 (1.98-1.95)	30.75-2.00 (2.03-2.00)
Unique reflections	34426 (1703)	32847 (1610)	31358 (1568)	29904 (1473)	27776 (1360)
Overall redundancy	13.7 (12.9)	13.8 (13.4)	13.8 (14.0)	13.8 (14.0)	13.8 (14.0)
Completeness, (%)	99.7 (99.9)	99.7 (99.9)	99.7 (99.8)	99.7 (99.9)	99.7 (99.9)
CC(1/2)	0.999 (0.485)	0.999 (0.520)	0.999 (0.518)	0.999 (0.562)	0.999 (0.497)
R_{pim} (I)	0.039 (0.806)	0.041 (0.803)	0.044 (0.804)	0.047 (0.777)	0.050 (0.754)
⟨I/σ(I)⟩	15.9 (1.0)	15.5 (1.1)	15.0 (1.0)	14.3 (1.1)	13.9 (1.1)
Wilson *B* value (Å^2)	24.87	25.66	26.40	27.05	28.11
DWD (kGy)	127.45	132.44	137.44	142.44	147.44

continues on the next page…

Supplementary Table S2 (part 7 of 7). Data collection and processing statistics [*CC*(1/2) = 0.5 cut-off criterion] for the experiment at RT.

…continues from the previous page

Data set	31	32	33	34	35
Beam Line	P14 (DESY)				
Beam shape	Top-hat				
Flux (photons/s)	3.65E+12				
Exposure time per image (s)	0.0752				
Δφ per image (°)	1				
Total exposure time per dataset (s)	2.7072				
Collimation (μm)	902 horizontal / 748 vertical				
Crystal size (μm)	750 × 650 × 550				
Wavelength (Å)	0.97625				
Temperature (K)	293				
Space group	*I*222				
Cell dimensions (Å) *a b c*	79.66 96.52 105.58	79.65 96.50 105.55	79.63 96.49 105.50	79.57 96.48 105.42	79.71 96.41 105.47
Resolution range (Å) Highest res. bin (Å)	30.75-2.06 (2.10-2.06)	30.75-2.14 (2.18-2.14)	30.75-2.25 (2.29-2.25)	30.75-2.47 (2.51-2.47)	30.75-2.57 (2.61-2.57)
Unique reflections	25458 (1287)	22728 (1103)	19578 (943)	14860 (724)	13247 (641)
Overall redundancy	13.7 (14.0)	13.7 (13.9)	13.7 (13.9)	13.6 (13.8)	13.6 (13.7)
Completeness, (%)	99.7 (99.8)	99.7 (99.8)	99.7 (99.9)	99.7 (99.9)	99.7 (99.7)
CC(1/2)	0.998 (0.546)	0.998 (0.516)	0.997 (0.440)	0.995 (0.362)	0.992 (0.402)
R_{pim} (I)	0.055 (0.744)	0.062 (0.707)	0.072 (0.766)	0.086 (0.812)	0.100 (0.798)
⟨I/σ(I)⟩	13.0 (1.1)	12.0 (1.2)	11.1 (1.1)	9.5 (1.1)	7.9 (1.1)
Wilson *B* value ($Å^2$)	29.30	30.28	31.62	34.12	33.57
DWD (kGy)	152.44	157.43	163.43	167.43	172.43

Supplementary Table S3 (part 1 of 5). Data collection and processing statistics [resolution = 1.84 Å cut-off criterion] for the experiment at RT.

Data set	1	2	3	4	5
Beam Line	P14 (DESY)				
Beam shape	Top-hat				
Flux (photons/s)	3.65E+12				
Exposure time per image (s)	0.0752				
Δφ per image (°)	1				
Total exposure time per dataset (s)	2.7072				
Collimation (µm)	902 horizontal / 748 vertical				
Crystal size (µm)	750 × 650 × 550				
Wavelength (Å)	0.97625				
Temperature (K)	293				
Space group	*I*222				
Cell dimensions (Å) *a b c*	80.10 96.24 105.50	80.08 96.25 105.51	80.06 96.25 105.52	80.04 96.26 105.53	80.02 96.27 105.55
Resolution range (Å) Highest res. bin (Å)	30.00-1.84 (1.87-1.84)	30.00-1.84 (1.87-1.84)	30.00-1.84 (1.87-1.84)	30.00-1.84 (1.87-1.84)	30.00-1.84 (1.87-1.84)
Unique reflections	35563 (1729)	35562 (1726)	35559 (1724)	35570 (1733)	35571 (1735)
Overall redundancy	13.6 (12.6)	13.6 (12.5)	13.6 (12.5)	13.6 (12.5)	13.6 (12.5)
Completeness, (%)	99.6 (98.0)	99.6 (98.1)	99.6 (98.2)	99.6 (98.6)	99.6 (98.5)
CC(1/2)	0.999 (0.961)	0.999 (0.955)	0.999 (0.951)	0.999 (0.952)	0.999 (0.949)
R_{pim} (I)	0.019 (0.139)	0.019 (0.144)	0.019 (0.146)	0.019 (0.152)	0.020 (0.158)
⟨I/σ(I)⟩	29.9 (6.0)	29.8 (5.8)	29.5 (5.7)	29.3 (5.5)	28.9 (5.3)
Wilson *B* value (Å^2)	17.88	18.02	18.15	18.36	18.59
DWD (kGy)	2.50	7.50	12.49	17.49	22.49

continues on the next page…

Supplementary Table S3 (part 2 of 5). Data collection and processing statistics [resolution = 1.84 Å cut-off criterion] for the experiment at RT.

…continues from the previous page

Data set	6	7	8	9	10
Beam Line	P14 (DESY)				
Beam shape	Top-hat				
Flux (photons/s)	3.65E+12				
Exposure time per image (s)	0.0752				
Δφ per image (°)	1				
Total exposure time per dataset (s)	2.7072				
Collimation (μm)	902 horizontal / 748 vertical				
Crystal size (μm)	750 × 650 × 550				
Wavelength (Å)	0.97625				
Temperature (K)	293				
Space group	*I*222				
Cell dimensions (Å) *a b c*	80.01 96.27 105.56	80.00 96.28 105.57	79.99 96.28 105.58	79.98 96.29 105.58	79.97 96.30 105.59
Resolution range (Å) Highest res. bin (Å)	30.00-1.84 (1.87-1.84)	30.00-1.84 (1.87-1.84)	30.00-1.84 (1.87-1.84)	30.00-1.84 (1.87-1.84)	30.00-1.84 (1.87-1.84)
Unique reflections	35567 (1734)	35564 (1725)	35571 (1735)	35574 (1734)	35574 (1732)
Overall redundancy	13.6 (12.5)	13.6 (12.5)	13.6 (12.5)	13.6 (12.5)	13.6 (12.5)
Completeness, (%)	99.6 (98.5)	99.6 (98.1)	99.6 (98.6)	99.6 (98.6)	99.6 (98.6)
CC(1/2)	0.999 (0.946)	0.999 (0.944)	0.999 (0.935)	0.999 (0.929)	0.999 (0.923)
R_{pim} (I)	0.020 (0.164)	0.020 (0.171)	0.020 (0.176)	0.021 (0.186)	0.021 (0.195)
⟨I/σ(I)⟩	28.7 (5.1)	28.1 (4.9)	27.8 (4.7)	27.5 (4.5)	27.0 (4.3)
Wilson *B* value (Å^2)	18.75	18.95	19.17	19.40	19.68
DWD (kGy)	27.49	32.48	37.48	42.48	47.48

continues on the next page…

Supplementary Table S3 (part 3 of 5). Data collection and processing statistics [resolution = 1.84 Å cut-off criterion] for the experiment at RT.

…continues from the previous page

Data set	11	12	13	14	15
Beam Line	P14 (DESY)				
Beam shape	Top-hat				
Flux (photons/s)	3.65E+12				
Exposure time per image (s)	0.0752				
Δφ per image (°)	1				
Total exposure time per dataset (s)	2.7072				
Collimation (μm)	902 horizontal / 748 vertical				
Crystal size (μm)	750 × 650 × 550				
Wavelength (Å)	0.97625				
Temperature (K)	293				
Space group	*I*222				
Cell dimensions (Å) *a b c*	79.96 96.31 105.60	79.95 96.32 105.60	79.94 96.32 105.60	79.93 96.33 105.60	79.91 96.34 105.60
Resolution range (Å) Highest res. bin (Å)	30.00-1.84 (1.87-1.84)	30.00-1.84 (1.87-1.84)	30.00-1.84 (1.87-1.84)	30.00-1.84 (1.87-1.84)	30.00-1.84 (1.87-1.84)
Unique reflections	35579 (1737)	35578 (1727)	35572 (1729)	35566 (1729)	35566 (1726)
Overall redundancy	13.6 (12.5)	13.6 (12.5)	13.6 (12.5)	13.6 (12.5)	13.6 (12.5)
Completeness, (%)	99.6 (98.6)	99.6 (98.6)	99.6 (98.6)	99.6 (98.7)	99.6 (98.5)
CC(1/2)	0.999 (0.919)	0.999 (0.911)	0.999 (0.903)	0.999 (0.889)	0.999 (0.884)
R_{pim} (I)	0.022 (0.206)	0.022 (0.219)	0.022 (0.229)	0.023 (0.248)	0.024 (0.267)
⟨I/σ(I)⟩	26.3 (4.1)	25.8 (3.9)	25.4 (3.6)	24.7 (3.4)	24.3 (3.2)
Wilson *B* value ($Å^2$)	19.93	20.24	20.54	20.92	21.23
DWD (kGy)	52.47	57.47	62.47	67.47	72.47

continues on the next page…

Supplementary Table S3 (part 4 of 5). Data collection and processing statistics [resolution = 1.84 Å cut-off criterion] for the experiment at RT.

…continues from the previous page

Data set	16	17	18	19	20
Beam Line	P14 (DESY)				
Beam shape	Top-hat				
Flux (photons/s)	3.65E+12				
Exposure time per image (s)	0.0752				
Δφ per image (°)	1				
Total exposure time per dataset (s)	2.7072				
Collimation (μm)	902 horizontal / 748 vertical				
Crystal size (μm)	750 × 650 × 550				
Wavelength (Å)	0.97625				
Temperature (K)	293				
Space group	*I*222				
Cell dimensions (Å) *a b c*	79.90 96.35 105.60	79.89 96.36 105.61	79.87 96.38 105.61	79.85 96.38 105.61	79.83 96.39 105.61
Resolution range (Å) Highest res. bin (Å)	30.00-1.84 (1.87-1.84)	30.00-1.84 (1.87-1.84)	30.00-1.84 (1.87-1.84)	30.00-1.84 (1.87-1.84)	30.00-1.84 (1.87-1.84)
Unique reflections	35562 (1729)	35562 (1727)	35564 (1727)	35551 (1727)	35551 (1743)
Overall redundancy	13.6 (12.5)	13.6 (12.6)	13.6 (12.6)	13.6 (12.5)	13.6 (12.5)
Completeness, (%)	99.6 (98.2)	99.6 (98.1)	99.6 (98.4)	99.6 (98.2)	99.6 (99.0)
CC(1/2)	0.999 (0.853)	0.999 (0.843)	0.999 (0.812)	0.999 (0.777)	0.999 (0.773)
R_{pim} (I)	0.024 (0.286)	0.025 (0.310)	0.026 (0.341)	0.027 (0.376)	0.028 (0.414)
⟨I/σ(I)⟩	23.6 (2.9)	23.0 (2.7)	22.3 (2.5)	21.6 (2.2)	20.9 (2.0)
Wilson *B* value ($Å^2$)	21.57	21.89	22.26	22.59	22.94
DWD (kGy)	77.46	82.46	87.46	92.46	97.46

continues on the next page…

Supplementary Table S3 (part 5 of 5). Data collection and processing statistics [resolution = 1.84 Å cut-off criterion] for the experiment at RT.

…continues from the previous page

Data set	21	22	23	24	25
Beam Line	P14 (DESY)				
Beam shape	Top-hat				
Flux (photons/s)	3.65E+12				
Exposure time per image (s)	0.0752				
Δφ per image (°)	1				
Total exposure time per dataset (s)	2.7072				
Collimation (μm)	902 horizontal / 748 vertical				
Crystal size (μm)	750 × 650 × 550				
Wavelength (Å)	0.97625				
Temperature (K)	293				
Space group	*I*222				
Cell dimensions (Å) *a b c*	79.82 96.40 105.61	79.80 96.41 105.61	79.78 96.43 105.61	79.77 96.45 105.61	79.75 96.46 105.61
Resolution range (Å) Highest res. bin (Å)	30.00-1.84 (1.87-1.84)	30.00-1.84 (1.87-1.84)	30.00-1.84 (1.87-1.84)	30.00-1.84 (1.87-1.84)	30.00-1.84 (1.87-1.84)
Unique reflections	35551 (1746)	35556 (1739)	35561 (1741)	35557 (1738)	35557 (1738)
Overall redundancy	13.6 (12.5)	13.6 (12.4)	13.6 (12.4)	13.6 (12.4)	13.6 (12.4)
Completeness, (%)	99.6 (99.1)	99.6 (98.8)	99.7 (99.4)	99.7 (99.0)	99.7 (98.9)
CC(1/2)	0.999 (0.704)	0.999 (0.650)	0.999 (0.599)	0.999 (0.566)	0.999 (0.466)
R_{pim} (I)	0.029 (0.471)	0.031 (0.511)	0.032 (0.596)	0.034 (0.691)	0.037 (0.757)
⟨I/σ(I)⟩	20.1 (1.8)	19.4 (1.6)	18.5 (1.4)	17.6 (1.2)	16.6 (1.1)
Wilson *B* value ($Å^2$)	23.22	23.57	23.82	24.11	24.25
DWD (kGy)	102.45	107.45	112.45	117.45	122.45

Supplementary Table S4. 5-PMUA occupancy values from multiple *Servalcat* refinement runs for the RT experiment.

For occupancy (q) refinement, we applied the constraint: $q_1 + q_2 = 1$ with q(5-PMUA) = q_1 and q(MUA•) = q(W1) = q_2. Values for q_2 are therefore 1- q_1.

Dose (kGy)	q_1 (starting q_1=0.1/ q_2=0.9)	q_1 (starting q_1=0.3/ q_2=0.7)	q_1 (starting q_1=0.5/ q_2=0.5)	q_1 (starting q_1=0.7/ q_2=0.3)	q_1 (starting q_1=0.9/ q_2=0.1)
2.498	0.65	0.65	0.66	0.66	0.67
7.496	0.59	0.60	0.60	0.61	0.62
12.493	0.50	0.51	0.51	0.52	0.53
17.491	0.42	0.43	0.44	0.46	0.49
22.488	0.34	0.35	0.35	0.36	0.36
27.486	0.26	0.27	0.28	0.29	0.30
32.483	0.19	0.20	0.20	0.21	0.22
37.481	0.06	0.06	0.07	0.08	0.09
42.479	0.09	0.10	0.10	0.11	0.11
47.477	0.03	0.03	0.05	0.07	0.09
52.475	0.01	0.00	0.01	0.02	0.02
57.473	0.01	0.01	0.02	0.03	0.05
62.471	0.05	0.06	0.06	0.06	0.07
67.469	0.02	0.02	0.02	0.03	0.03
72.467	0.04	0.04	0.05	0.05	0.05

Supplementary Table S5. 5-PMUA occupancy values from *a* single refinement run with *phenix.refine* for the RT experiment.

For occupancy (q) refinement, we applied the constraint: $q_1 + q_2 = 1$ with q(5-PMUA) = q_1 and q(MUA•) = q(W1) = q_2. Values for q_2 are therefore 1- q_1.

Dose (kGy)	q_1 (random starting values for q_1 and q_2)
2.498	0.72
7.496	0.70
12.493	0.54
17.491	0.49
22.488	0.46
27.486	0.38
32.483	0.28
37.481	0.21
42.479	0.16
47.477	0.13
52.475	0.12
57.473	0.12
62.471	0.10
67.469	0.12
72.467	0.09

Supplementary Table S6 (part 1 of 4). Occupancy values from multiple *Servalcat* refinement runs for the experiment at 100 K.
For occupancy (q) refinement, we applied the constraints: q(5-PMUA)+q(MUA_T)=1, q(5-PMUA)+q(O_2)+q(W1)=1, q(MUA_T)=q(O_2)+q(W1).

Dose (kGy)	q(5-PMUA) starting q(5-PMUA)=0.90 q(MUA_T)=0.10 q(O_2)=0.05 q(W1)=0.05	q(5-PMUA) starting q(5-PMUA)=0.75 q(MUA_T)=0.25 q(O_2)=0.20 q(W1)=0.05	q(5-PMUA) starting q(5-PMUA)=0.50 q(MUA_T)=0.50 q(O_2)=0.45 q(W1)=0.05	q(5-PMUA) starting q(5-PMUA)=0.25 q(MUA_T)=0.75 q(O_2)=0.70 q(W1)=0.05	q(5-PMUA) starting q(5-PMUA)=0.10 q(MUA_T)=0.90 q(O_2)=0.85 q(W1)=0.05
2.340	0.83	0.82	0.80	0.79	0.79
11.726	0.76	0.75	0.73	0.72	0.72
21.106	0.74	0.73	0.71	0.69	0.69
30.486	0.65	0.64	0.63	0.61	0.61
39.866	0.60	0.59	0.58	0.56	0.55
58.626	0.52	0.52	0.50	0.50	0.49
77.386	0.48	0.47	0.46	0.44	0.44
96.146	0.42	0.41	0.40	0.39	0.38
128.976	0.36	0.35	0.33	0.32	0.32
161.806	0.33	0.32	0.31	0.30	0.30
208.706	0.26	0.25	0.24	0.23	0.23
260.296	0.22	0.22	0.21	0.20	0.20
321.266	0.19	0.19	0.18	0.17	0.17
372.856	0.16	0.16	0.15	0.14	0.14

continues on the next page…

Supplementary Table S6 (part 2 of 4). Occupancy values from multiple *Servalcat* refinement runs for the experiment at 100 K.

…continues from the previous page

Dose (kGy)	$q(MUA_T)$ starting q(5-PMUA)=0.90 $q(MUA_T)$=0.10 $q(O_2)$=0.05 q(W1)=0.05	$q(MUA_T)$ starting q(5-PMUA)=0.75 $q(MUA_T)$=0.25 $q(O_2)$=0.20 q(W1)=0.05	$q(MUA_T)$ starting q(5-PMUA)=0.50 $q(MUA_T)$=0.50 $q(O_2)$=0.45 q(W1)=0.05	$q(MUA_T)$ starting q(5-PMUA)=0.25 $q(MUA_T)$=0.75 $q(O_2)$=0.70 q(W1)=0.05	$q(MUA_T)$ starting q(5-PMUA)=0.10 $q(MUA_T)$=0.90 $q(O_2)$=0.85 q(W1)=0.05
2.340	0.17	0.18	0.20	0.21	0.21
11.726	0.24	0.25	0.27	0.28	0.28
21.106	0.26	0.27	0.29	0.30	0.31
30.486	0.35	0.36	0.37	0.39	0.39
39.866	040	0.41	0.42	0.44	0.45
58.626	0.48	0.48	0.50	0.50	0.51
77.386	0.52	0.53	0.54	0.56	0.56
96.146	0.58	0.59	0.60	0.61	0.62
128.976	0.64	0.65	0.67	0.68	0.69
161.806	0.67	0.68	0.69	0.70	0.70
208.706	0.74	0.75	0.76	0.77	0.77
260.296	0.78	0.78	0.79	0.80	0.80
321.266	0.81	0.81	0.82	0.83	0.83
372.856	0.84	0.84	0.85	0.86	0.86

continues on the next page…

Supplementary Table S6 (part 3 of 4). Occupancy values from multiple *Servalcat* refinement runs for the experiment at 100 K.

…continues from the previous page

Dose (kGy)	$q(O_2)$ starting q(5-PMUA)=0.90 $q(MUA_T)$=0.10 $q(O_2)$=0.05 q(W1)=0.05	$q(O_2)$ starting q(5-PMUA)=0.75 $q(MUA_T)$=0.25 $q(O_2)$=0.20 q(W1)=0.05	$q(O_2)$ starting q(5-PMUA)=0.50 $q(MUA_T)$=0.50 $q(O_2)$=0.45 q(W1)=0.05	$q(O_2)$ starting q(5-PMUA)=0.25 $q(MUA_T)$=0.75 $q(O_2)$=0.70 q(W1)=0.05	$q(O_2)$ starting q(5-PMUA)=0.10 $q(MUA_T)$=0.90 $q(O_2)$=0.85 q(W1)=0.05
2.340	0.09	0.09	0.09	0.09	0.09
11.726	0.16	0.16	0.17	0.17	0.17
21.106	0.19	0.19	0.20	0.20	0.20
30.486	0.28	0.28	0.29	0.29	0.30
39.866	0.35	0.35	0.35	0.36	0.36
58.626	0.42	0.42	0.43	0.43	0.43
77.386	0.43	0.43	0.44	0.45	0.45
96.146	0.50	0.50	0.51	0.51	0.52
128.976	0.56	0.57	0.57	0.58	0.58
161.806	0.61	0.61	0.62	0.62	0.62
208.706	0.68	0.68	0.68	0.69	0.69
260.296	0.69	0.69	0.69	0.68	0.68
321.266	0.73	0.73	0.73	0.73	0.73
372.856	0.75	0.75	0.75	0.75	0.75

continues on the next page…

Supplementary Table S6 (part 4 of 4). Occupancy values from multiple *Servalcat* refinement runs for the experiment at 100 K.

…continues from the previous page

Dose (kGy)	q(W1) starting q(5-PMUA)=0.90 $q(MUA_T)$=0.10 $q(O_2)$=0.05 q(W1)=0.05	q(W1) starting q(5-PMUA)=0.75 $q(MUA_T)$=0.25 $q(O_2)$=0.20 q(W1)=0.05	q(W1) starting q(5-PMUA)=0.50 $q(MUA_T)$=0.50 $q(O_2)$=0.45 q(W1)=0.05	q(W1) starting q(5-PMUA)=0.25 $q(MUA_T)$=0.75 $q(O_2)$=0.70 q(W1)=0.05	q(W1) starting q(5-PMUA)=0.10 $q(MUA_T)$=0.90 $q(O_2)$=0.85 q(W1)=0.05
2.340	0.08	0.09	0.10	0.11	0.12
11.726	0.08	0.09	0.10	0.11	0.11
21.106	0.07	0.08	0.09	0.10	0.11
30.486	0.07	0.08	0.09	0.09	010
39.866	0.05	0.06	0.07	0.08	0.08
58.626	0.06	0.06	0.07	0.08	0.08
77.386	0.09	0.10	0.10	0.11	0.11
96.146	0.08	0.09	0.09	0.10	0.10
128.976	0.08	0.09	0.09	0.10	0.10
161.806	0.06	0.07	0.07	0.08	0.08
208.706	0.07	0.07	0.07	0.08	0.08
260.296	0.09	0.09	0.10	0.11	0.12
321.266	0.08	0.08	0.09	0.10	0.11
372.856	0.09	0.10	0.10	0.11	0.11

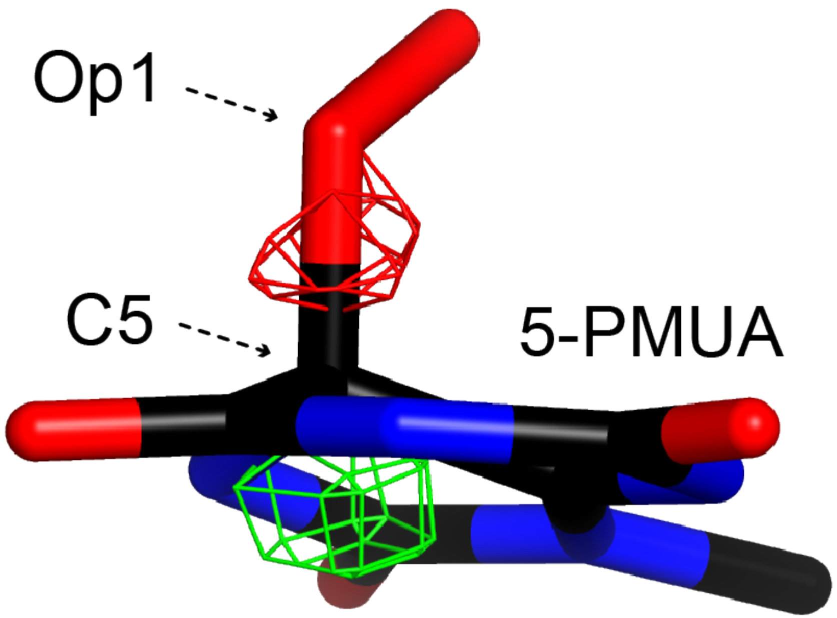

Supplementary Figure S1. 5-PMUA is already partly damaged in the low-dose (2.50 kGy) RT experiment. Occupancy refinement for 5-PMUA converged to 1.0 and 0.94 using *Servalcat* and *phenix.refine*. However, clear negative difference density along the C5—Op1 bond and positive difference density indicating loss of pyramidalization at C5 together suggest the presence of both 5-PMUA and MUA•. Fourier difference maps are shown as mesh representation at the +3σ and -3σ levels in green and red, respectively.

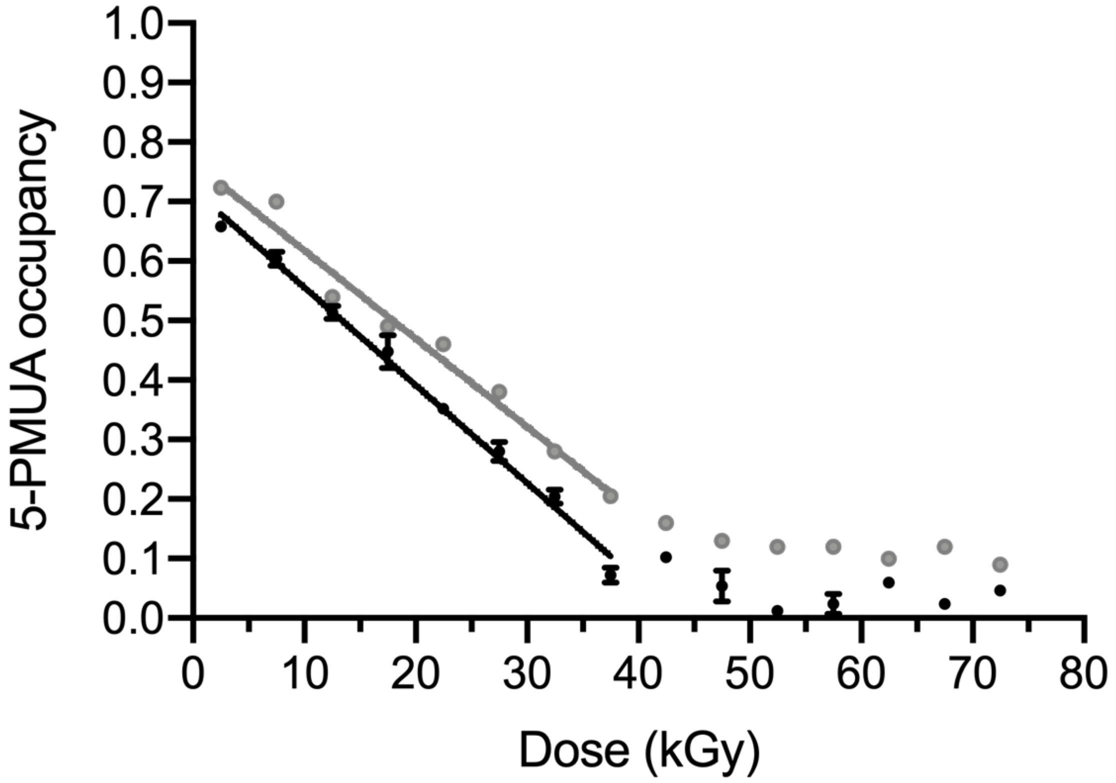

Supplementary Figure S2. Dose-dependent 5-PMUA decay at RT. 5-PMUA occupancy derived from *Servalcat* (black dots) and from *phenix.refine* (grey dots) refinement. Values reported for *Servalcat* are the average of five independent refinement runs initialised at different occupancy values. Error bars are standard deviations. Values from *phenix.refine* are the result of a single run initialised at random occupancy values. Both packages indicate that 5-PMUA decays linearly with dose at the similar rate of -0.016 (*Servalcat*) and -0.015 (*phenix.refine*).

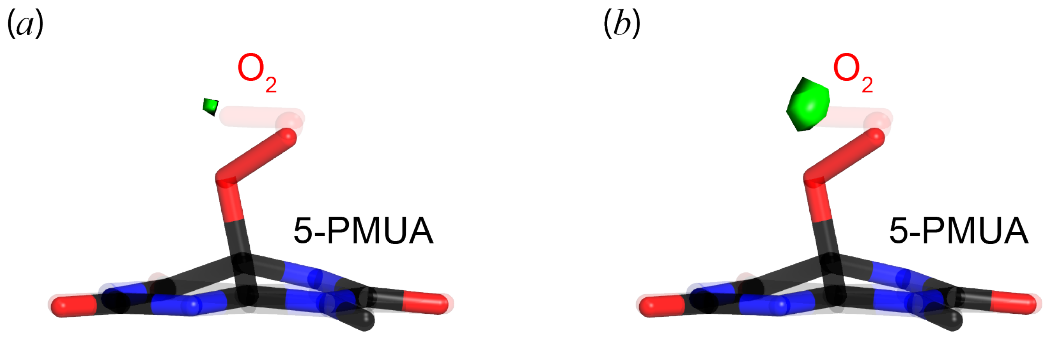

Supplementary Figure S3. Minor signs of radiolysis are already present in the low-dose datasets at 100 K. Fourier difference maps are shown as isosurfaces at the +3σ level in green for the (*a*) 2.34 kGy and (*b*) 11.7 kGy datasets. Positive density is localised at the position where dioxygen is released. The O_2 molecule is shown as transparent stick for reference.

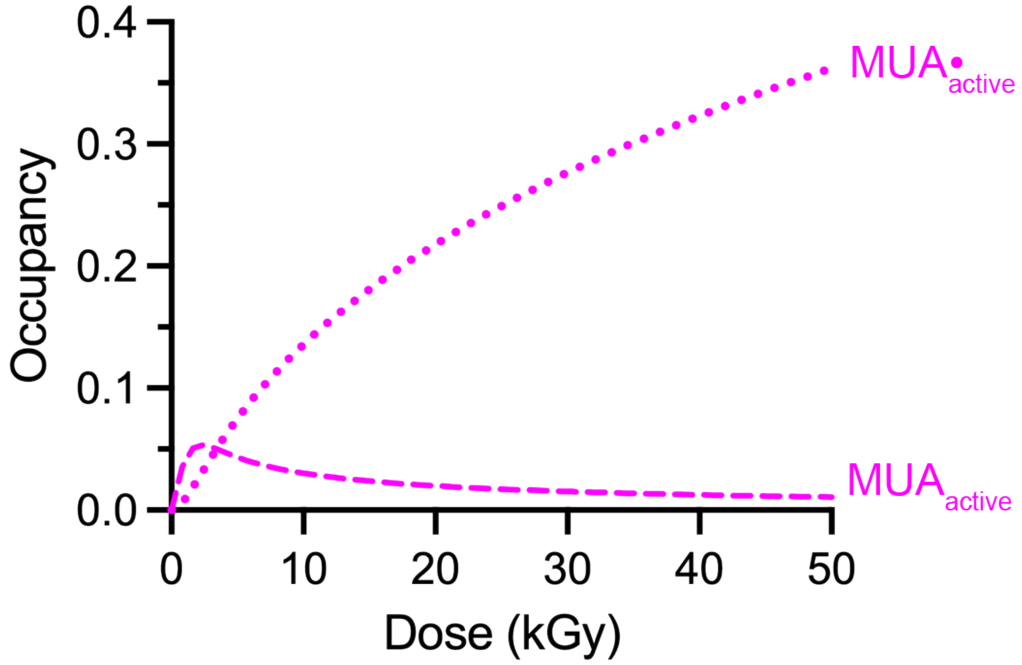

Supplementary Figure S4. Occupancy profiles of MUA_{active} and $MUA\bullet_{active}$ in the dose region up to 50 kGy as derived from the kinetic fitting of the scheme in Figure 6b of the main text.

(*a*)

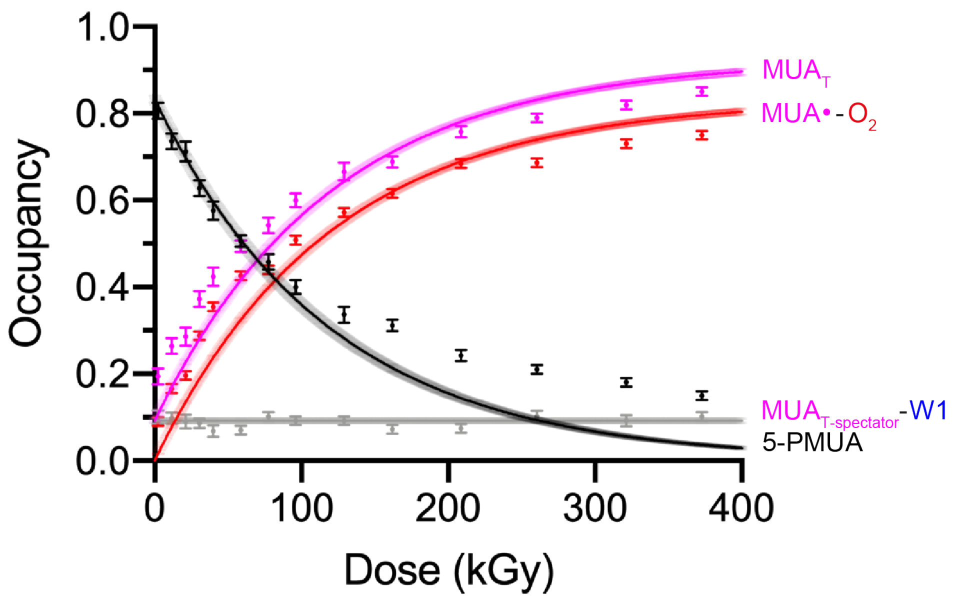

(*b*)

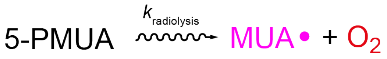

Supplementary Figure S5. A simple radiolytic mechanism completely fails to explain dose-dependent occupancies for the 100 K experiment. (*a*) Occupancy values from *Servalcat* refinement for the species indicated in the legend are shown as coloured circles with error bars corresponding to their standard deviations calculated from multiple refinement rounds as indicated in the main text. The curves represent the kinetic fit according to the scheme shown in (*b*). Transparent error bars are lower and upper confidence intervals. (*b*) Kinetic scheme employed to model the experimental occupancies.
